
JOURNAL INFORMATION
==============================
NLM Title Abbreviation: J Mol Cell Biol
Journal ID: J Mol Cell Biol
NLM Journal ID: 101503669
Journal ID: jmcb

Journal of Molecular Cell Biology

ISSN: 1674-2788
EISSN: 1759-4685

ARTICLE INFORMATION
==============================
PMCID: PMC8574307
PMID: 34687317
DOI: 10.1093/jmcb/mjab068
Article ID: mjab068
Article version: 1
Subjects: Letter to the Editor, AcademicSubjects/SCI01180

Interactome profiling reveals interaction of SARS-CoV-2 NSP13 with host factor STAT1 to suppress interferon signaling

Feng Kuan 1 2
Min Yuan-Qin 1 3
Sun Xiulian 1 3
Deng Fei 1 3
Li Peiqing 2
Wang Hualin 1 3
Ning Yun-Jia 1 3
1 State Key Laboratory of Virology, Wuhan Institute of Virology, Chinese Academy of Sciences , Wuhan, 430071 China
2 Department of Pediatric Emergency, Guangzhou Women and Children’s Medical Center, Guangzhou Medical University , Guangzhou, 510623 China
3 Center for Biosafety Mega-Science, Chinese Academy of Sciences , Wuhan, 430071 China
These authors contributed equally to this work.

Correspondence to: Hualin Wang, E-mail: h.wang@wh.iov.cn Yun-Jia Ning, E-mail: nyj@wh.iov.cn Peiqing Li, E-mail: annie_129@126.com
Electronic publication date: 2021 Oct 23
Electronic Location ID: mjab068
Received 2021 Apr 24; Revised 2021 Sep 26; Accepted 2021 Sep 28
Copyright: © The Author(s) 2021. Published by Oxford University Press on behalf of Journal of Molecular Cell Biology, IBCB, SIBS, CAS.
Copyright year: 2021
License: This is an Open Access article distributed under the terms of the Creative Commons Attribution License (https://creativecommons.org/licenses/by/4.0/), which permits unrestricted reuse, distribution, and reproduction in any medium, provided the original work is properly cited.
License URL: https://creativecommons.org/licenses/by/4.0/

Keywords: SARS-CoV-2, COVID-19, coronavirus, NSP13, immune evasion, interferon, STAT1, interactome, druggable target
Page count: 15
edited-state: accepted-manuscript
article-lifecycle: PAP

==============================

Kuan Feng[1,2,†], Yuan-Qin Min[1,3,†], Xiulian Sun[1,3], Fei Deng[1,3], Peiqing Li[2,*], Hualin Wang[1,3,*], Yun-Jia Ning[1,3,*]

**Running title:** SARS-CoV-2 NSP13 interactome and IFN signaling antagonism.

**Dear Editor,**

The on-going coronavirus disease 2019 (COVID-19) pandemic caused by severe acute respiratory syndrome coronavirus 2 (SARS-CoV-2) has resulted in unprecedented medical and socioeconomic disruption globally. As of late September 2021, over 231 million confirmed cases have been reported worldwide. Although the viral pathogenesis remains largely unclear, impairment of interferon (IFN) responses likely contributes to disease progression and severity (Blanco-Melo et al., 2020; Meffre and Iwasaki, 2020; Min et al., 2021). Indeed, several viral proteins are potential regulators of the IFN system (Min et al., 2021). However, the underlying mechanisms employed by SARS-CoV-2 IFN antagonist candidates, such as nonstructural protein 13 (NSP13), still need to be determined.

NSP13 is important in viral replication as a helicase unwinding duplex RNA and a 5′-triphosphatase likely involved in 5′-capping of viral mRNA (Min et al., 2021). We observed robust inhibition of type I IFN signaling by SARS-CoV-2 NSP13 through reporter gene assays (Figure 1A), consistent with previous observations (Lei et al., 2020; Yuen et al., 2020; Xia et al., 2020), highlighting the versatile activities of NSP13 in viral infection. Furthermore, NSP13 inhibition of type I IFN signaling was confirmed by qPCR analyses of the induction of antiviral IFN-stimulated genes (ISGs) (Supplementary Figure S1A). Although Gordon et al. (2020) recently reported possible host molecules interacting with SARS-CoV-2 proteins, none of the 40 cellular proteins identified with NSP13 as bait are involved in IFN-triggered signaling pathways. Additionally, time-sensitive preparation of the manuscript precluded visual results (immunoblotting or silver staining) demonstrating effective precipitation of baits and cellular proteins before mass spectrometry (MS) (Gordon et al., 2020). Thus, further exploration of the viral interactome is worthwhile.

To elucidate the mechanism of NSP13-mediated IFN signaling antagonism and further decipher the potential cellular interaction landscape of such an important viral protein, we exploited a high-affinity purification assay (S-pulldown) coupled with quantitative MS to identify the cellular interactome of NSP13 (Figure 1B). S-tagged NSP13 expressed in cells was efficiently precipitated, and bands of cellular proteins co-precipitating with NSP13 were also evident (Figure 1C and D). MS analysis identified 172 cellular proteins as potential NSP13-interacting host factors with high confidence (Supplementary Table S1 and Figure S2A); 171 were newly identified in this study (C1orf50 was also reported by Gordon et al., 2020). These proteins might affect NSP13 function or be affected by NSP13. We next validated the interactions of NSP13 with a series of representative host proteins through immunoblotting (Supplementary Figure S3), further supporting the new interactome. Gene ontology and protein–protein interaction network analyses showed that these host proteins were enriched in several important biological processes and some specific molecular functions and cellular components (Figure 1E and F; Supplementary Figure S2D and E), highlighting novel and possibly significant aspects of SARS-CoV-2 biology. For instance, enrichment of NSP13-interacting proteins in regulation of mRNA stability might be linked to the role of NSP13 in viral mRNA capping. Interactions of NSP13 with host proteins involved in IL-1 signaling and MHC-I antigen presentation may modulate these pivotal inflammatory and immune responses during SARS-CoV-2 infection. Intriguingly, the NSP13 interactome was enriched in proteins associated with transcriptional regulation in response to hypoxia (a pivotal and concerning pathophysiological feature of COVID-19 pneumonia; Kashani, 2020), indicating novel potential roles of NSP13 in COVID-19 pathogenesis that merit future exploration. Furthermore, we identified 45 pharmacological cellular targets along with 95 corresponding drugs by drug database mining with the interactome (Figure 1G; Supplementary Table S2), revealing potentially valuable clues for future design of a NSP13–host interface-directed intervention strategy.

Importantly, an essential component of the IFN-triggered signaling pathway, STAT1 (Najjar and Fagard, 2010), was identified as a potential NSP13-interacting protein (Supplementary Table S1 and Figure S2A–C). Given the central role of STAT1 in IFN signaling cascades (Najjar and Fagard, 2010), we speculated that STAT1 might be the cellular interaction target of NSP13 for IFN signaling counteraction. Immunoblotting indicated that STAT1 indeed co-precipitated with NSP13 (Figure 1H). Moreover, reciprocal interaction analysis in the context of SARS-CoV-2 infection confirmed co-precipitation of NSP13 with STAT1 (Figure 1I). The NSP13–STAT1 interaction was further demonstrated to be independent of RNA or DNA using a nuclease treatment assay (Mo et al., 2020) before protein-interaction detection (Supplementary Figure S4). Then, we constructed a series of C-terminal truncations based on NSP13 3D structure to map the interaction domain (Figure 1J and K). Interestingly, truncated NSP13 with the 2A domain deleted (N442) retained interaction with STAT1, whereas removal of the 1A domain (N259) or further deletions (N150 or N100) abolished NSP13–STAT1 interaction (Figure 1L), indicating a necessary role of 1A domain (Figure 1J) in NSP13 targeting to STAT1. Furthermore, immunofluorescence assays (IFA) demonstrated that NSP13 expression blocks nuclear accumulation of STAT1 induced by IFN-$\alpha$ (Figure 1M). Interestingly, NSP13 mutant N259 failed to block STAT1 nuclear translocation, while mutant N442 retained inhibitory ability against STAT1 nuclear accumulation (Supplementary Figure S5), in line with their interactions with STAT1 (Figure 1L). IFN-$\alpha$-triggered STAT1 phosphorylation, a prerequisite for STAT1 activation and efficient nuclear translocation, was noticeably reduced by NSP13 (Figure 1N). Moreover, inhibition of IFN-$\alpha$-stimulated STAT1 activation by NSP13 was comparable to or stronger than that by SOCS1 (Supplementary Figure S6A), a well-known cellular negative regulator of Janus kinase (Najjar and Fagard, 2010). These consistent observations corroborate that NSP13 suppresses type I IFN signaling by interacting with STAT1 and hence impairing the host protein's actions. As STAT1 is an indispensable signaling molecule shared by all type I, II, and III IFN signaling cascades, we considered that NSP13 might antagonize type II and III IFN signaling as well. Indeed, NSP13 expression significantly inhibited both IFN-$\gamma$– and IFN-$\lambda$–induced reporter activation (Figure 1O and P) and antiviral ISG expression (Supplementary Figure S1B and C). Furthermore, STAT1 phosphorylation triggered by type II and III IFNs was also suppressed by NSP13 (Figure 1Q and R; Supplementary Figure S6B and C). Together, these data suggest that NSP13 targets STAT1 through interaction requiring the NSP13 1A domain, thus interfering with STAT1 actions. The interference ultimately inhibits type I, II, and III IFN signaling cascades that orchestrate complex host immune responses upon viral infection.

In summary, we uncovered a new interactome of the critical viral protein NSP13, revealing important aspects of SARS-CoV-2 biology as well as potential druggable targets and targeting drugs at the interface of virus–host interactions. We further demonstrated that nucleic acid-independent interaction of NSP13 with STAT1 involves NSP13 domain 1A and results in inhibition of type I, II, and III IFN-elicited STAT1 actions. Given the IFN-antagonistic and hence potential virulence-associated activities of NSP13, future studies of specific NSP13 mutant viruses (e.g. those constructed by reverse genetics) will further elucidate the biological significance of NSP13 targeting of STAT1 or other host factors and help inform the design of attenuated vaccines. Our findings unravel the cellular interaction target and mechanism for NSP13 blockade of IFN signaling and provide new insights into virus–host interactions from molecular and proteomic perspectives, which benefit not only better understanding of SARS-CoV-2 infection but also further development of antiviral therapies.

*[Supplementary material is available at Journal of Molecular Cell Biology online. We thank Dr Hong-Bing Shu (Wuhan University) for providing the reporter plasmids, Dr Xinwen Chen (Wuhan Institute of Virology) for the viral vector packaging plasmids, the National Virus Resource Center (Wuhan Institute of Virology) for virus resources, and the Core Facility and Technical Support of Wuhan Institute of Virology for technical assistance. We are also grateful to the administration team of the National Biosafety Laboratory, Wuhan, Chinese Academy of Sciences. This work was supported by grants from the National Natural Science Foundation of China (31870162 and 82161138003), the National Key Research and Development Program of China (2018YFA0507202), and the Youth Innovation Promotion Association of Chinese Academy of Sciences.]*

### Figure legend

**Figure 1** A new interactome of SARS-CoV-2 NSP13 reveals STAT1 as the cellular interaction target for NSP13 antagonism of IFN signaling cascades. (**A**) NSP13 suppresses type I IFN-triggered signaling. Dual-luciferase reporter (DLR) assays were conducted as described in Supplementary Materials and methods. Relative luciferase activity (Rel. luc. act) is shown. (**B**) Workflow for identifying cellular proteins interacting with NSP13. (**C** and **D**) Silver-stained SDS-polyacrylamide gel and immunoblot of control or NSP13 pulldown products. Red arrows indicate efficient precipitation of NSP13. (**E** and **F**) Biological process enrichment (**E**) and STRING analysis (**F**) for NSP13-interacting proteins (fold change >2, $P < 0.05$). The top 15 enriched gene ontologies are shown (**E**). (**G**) Drug–cellular target network. (**H**) Validation of NSP13–STAT1 interaction using S-pulldown (NSP13 as bait) and immunoblotting with HEK293T cells. (**I**) Reciprocal S-pulldown assay using HEK293-ACE2 cells infected with SARS-CoV-2, further corroborating NSP13–STAT1 interaction. S-pulldown assay was performed at 24 h postinfection. (**J**) 3D structure of NSP13 (PDB: 6ZSL). (**K**) Linear representation of domain organization in full-length or C-terminal-truncated NSP13 (fused with HA tag). (**L**) S-pulldown and immunoblotting showing interactions of truncated NSP13 proteins with STAT1 in HEK293T cells. (**M**) STAT1 nuclear translocation detected using confocal microscopy. At 24 h post-transfection, HEK293 cells transfected with control or HA-tagged NSP13 expression plasmids were treated with IFN-α (2000 u/ml) for 30 min before IFA with anti-HA or anti-STAT1 antibodies; >150 cells for each group were then scored for STAT1 nuclear translocation. Scale bar, 7 μm. (**N**) HEK293T cells transfected with vector or S-tagged NSP13 expression plasmid were left untreated or treated with IFN-α (2000 u/ml) for 30 min, followed by immunoblotting. (**O** and **P**) NSP13 inhibits signal transduction driven by type II (IFN-γ; **O**) and type III (IFN-λ; **P**) IFNs. DLR assays for IFN-γ and IFN-λ signaling were performed. (**Q** and **R**) Cells were treated as in **N**, except for replacement of IFN-α by IFN-γ (100 ng/ml) or IFN-λ (200 ng/ml). Data in histograms are mean ± SD, $n = 4$ (**A**, **O**, and **P**) or 3 (**M**). $^{****}P < 0.0001$, $^{***}P < 0.001$. Numbers beside gels or blots indicate molecular mass (kDa).

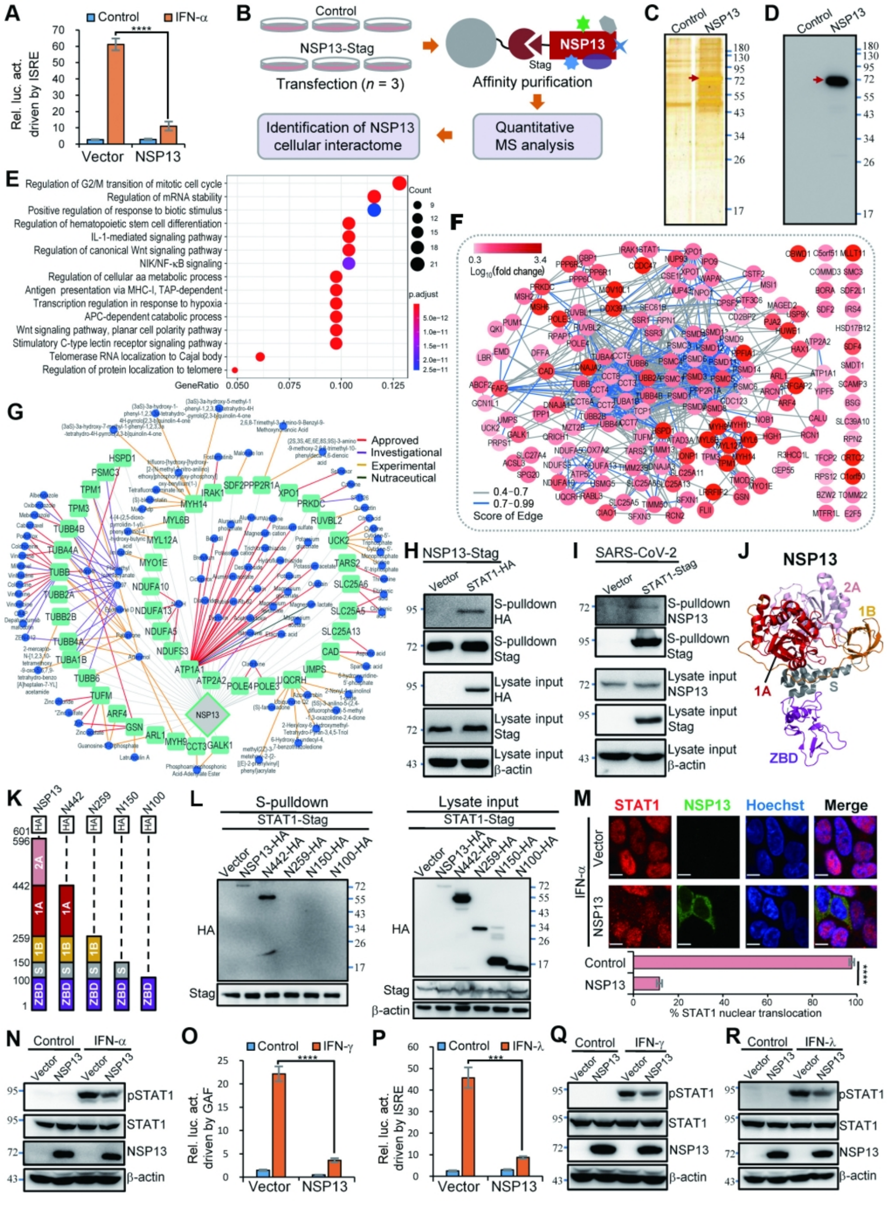

Figure 1. A new interactome of SARS-CoV-2 NSP13 reveals STAT1 as the cellular interaction target for NSP13 antagonism of IFN signaling cascades.

170x230mm (300 x 300 DPI)

## Supplementary materials and methods

### Cells and virus

HEK293 (ATCC, CRL-1573) and Vero E6 cells (ATCC, CRL-1586) were maintained in Eagle's minimum essential medium supplemented with 10% fetal bovine serum (FBS) at 37 °C in a humidified atmosphere with 5% $CO_2$. HEK293T (ATCC, CRL-11268) cells were cultured in Dulbecco's modified Eagle's medium. HEK293-ACE2 cell line was generated by transduction of lentiviral vector expressing ACE2 and subsequent monoclonal screening as described below. SARS-CoV-2 (nCoV-2019BetaCoV/Wuhan/WIV04/2019) was isolated from the clinical sample [1]. The propagation and titer determination of SARS-CoV-2 were performed in Vero E6 cells in a biosafety level-3 (BSL-3) laboratory of National Biosafety Laboratory, Wuhan, Chinese Academy of Sciences [1].

### Plasmids

Open reading frame (ORF) encoding nsp13 was amplified by RT-PCR from SARS-CoV-2 genomic RNA and cloned into the pCAGGSP7 vector without or with HA or S tag (Stag) [2-4]. Truncated nsp13 and SOCS1 expression plasmids were constructed into pCAGGSP7 vector with C-terminal HA tag by standard molecular biology methods. S or HA-tagged STAT1 expression plasmids were constructed as previously described [3, 5-7]. Human angiotensin I-converting enzyme-2 (ACE2) expression plasmid (pLVX-IRES-PURO-ACE2) was generated by cloning the ORF sequence of ACE2 into lentiviral vector pLVX-IRES-PURO. Luciferase reporter plasmids for type I, II, or III IFN-responsive promoters and the *Renilla* luciferase control plasmid (pRL-TK) were kindly provided by Prof. Hong-Bing Shu (Wuhan University) and were described previously [3, 6, 8-11].

### Antibodies and Reagents

Primary antibodies include mouse antibody to HA tag (Sigma-Aldrich, 05–904) and rabbit antibodies to S tag (Abcam, ab18588), β-actin (ABclonal, AC026), nsp13

(AtaGenix, ATP238), STAT1 (Abcam, ab109320), pSTAT1 (Cell Signaling Technology, 9167S), IRAK1 (proteintech, 10478-2-AP), HAX1 (proteintech, 11266-1-AP), DNAJA2 (ABclonal, A7059), HSPD1 (ABclonal, A0564), CCT4 (ABclonal, A6548), CCT8 (ABclonal, A4449), and TCP1 (ABclonal, A1950). For Immunofluorescence assay (IFA), secondary antibodies include Alexa Fluor 647-conjugated goat anti-rabbit IgG (H+L) (Abcam, ab150079) and Alexa Fluor 488-conjugated goat anti-mouse IgG (H+L) (Abcam, ab150113). For WB analysis, secondary antibodies include horseradish peroxidase (HRP)-conjugated goat anti-mouse or anti-rabbit IgG antibodies (Abcam, ab6789 and ab6721). Recombinant human IFN-α (300-02AA), IFN-γ (300-02), and IFN-λ (300-02K) were all purchased from Peprotech.

**Real-time qPCR**

Total RNA was extracted from the treated HEK293T cells with TRIzol reagent (NZK biotech, China). Reverse transcription of RNA was performed with a cDNA reverse transcription kit (TaKaRa, RR047A), followed by real-time quantitative PCR with an SYBR Green real-time PCR kit (TaKaRa, RR820A). The relative mRNA levels of the indicated genes were calculated using the $2^{-\Delta\Delta CT}$ method with *GAPDH* mRNA levels as an internal control. Primers used have been described in previous studies [12-15].

**Dual-luciferase reporter (DLR) gene assay**

HEK293T cells cultured in 96-well plates were cotransfected with 80 ng nsp13 expression plasmid or the control vector, together with 20 ng ISRE (for type I and III IFN signaling analyses) or GAF reporter plasmid (for type II IFN signaling analysis) and 0.5 ng pRL-TK, using lipofectamine 3000 (Invitrogen) by following the manufacturer’s instructions. At 24 h posttransfection, cells were mock stimulated or stimulated with IFN-α (type I IFN; 2000 u/ml), IFN-γ (type II IFN; 100 ng/ml), or IFN-λ (type III IFN; 200 ng/ml) for 16 h before measurements of luciferase activities with a DLR assay kit (Promega, E2940). The relative luciferase activities (Rel. Luc. Act) were exhibited by normalizing firefly luciferase activity to *Renilla* luciferase activity.

**Generation of HEK293-ACE2 cell line**

HEK293T cells were co-transfected with the human ACE2 expression plasmid pLVX-IRES-PURO-ACE2 and packaging plasmids provided by Prof. Xinwen Chen (Wuhan Institute of Virology) for 48 h [14, 16]. Packaged lentiviral particles were then harvested from culture medium and used for transduction of HEK293 cells, followed by 3-day selection with 2 μg/mL puromycin. Then, cells were diluted and seeded into 96-well plates for further screening of single colony stably over-expressing ACE2.

**Protein interaction, silver staining, and WB analyses**

S-pulldown assays utilizing the high-specific and strong affinity of S tag with S-protein coupled on agarose beads were performed as described previously to analyze protein interactions [3, 5, 14, 17-19]. In cotransfection assays before S-pulldown, HEK293T cells were transfected with the S-tagged protein expression plasmids together with the plasmids encoding other indicated proteins for 36 h. For protein interaction analysis in the contexts of infection combined with transfection, HEK293-ACE2 cells were firstly transfected with the plasmid encoding STAT1-Stag or the control vector and at 12 h posttransfection, the cells were infected with SARS-CoV-2 (MOI = 1) for 24 h. Then, the transfected or SARS-CoV-2-infected cells expressing S-tagged proteins were lysed with the immunoprecipitation lysis buffer (Beyotime) and after centrifugation, supernatants of cell lysates were mixed with S-protein agarose (Millipore) by rotating incubation. The beads were next washed with the lysis buffer and phosphate-buffered saline (PBS) successively, followed by silver staining, WB, or quantitative MS analyses of the bound protein samples. For silver staining analysis, protein samples were eluted with 1×SDS sample buffer by boiling for 10min and subjected to 10% SDS polyacrylamide gel electrophoresis (SDS-PAGE). Then, the gel was fixed, sensitized, and stained by Pierce Silver Stain Kit (Thermo Fisher Scientific) according to the manufacturer's instructions. For WB analysis, protein samples separated by SDS-PAGE were transferred onto polyvinylidene difluoride (PVDF) membranes. After blocking with 5% BSA in Tris-buffered saline-Tween 20 (TBS-T), the membranes were incubated successively with primary antibodies and HRP-conjugated secondary antibodies. Protein bands were detected with an enhanced chemiluminescence kit (Thermo Fisher Scientific). In nuclease treatment assays, cell lysates (supplemented with 2 mM MgCl2) were divided into halves and then subjected to excessive treatment with Benzonase (100 U, Millipore, Cat#70746) or left untreated at 4˚C for 2 h before S-pulldown as described previously [14].

**Quantitative MS analysis**

For MS quantification analysis, bead-bound proteins (in total of 6 samples from three biological replicates for both nsp13 pulldown and control groups) were denatured, reduced, alkylated, and eluted by one-step treatment in the reaction buffer (1% SDC/100 mM Tris-HCl, pH 8.5/10 mM TCEP/40 mM CAA) at 95 °C for 10 min. The eluates were diluted with equal volume of $H_2O$ and then subjected to trypsin digestion at 37 °C overnight. Obtained peptides were desalted for liquid chromatography-tandem mass spectrometry (LC-MS/MS) analysis using a Q Exactive HF-X system coupled with Easy-nLC 1200 (Thermo Fisher Scientific) [20]. MS raw tandem spectra were analyzed with MaxQuant (V1.6.6) (AB SCIEX) using the Andromeda database search algorithm [21]. Spectra files were searched against the Human Unified Protein Database (UniProt) [22]. The main parameters were as follows: LFQ mode was checked for quantification; Variable modifications, Oxidation (M) & Acetyl (Protein N-term); Fixed modifications, Carbamidomethyl (C); Digestion, Trypsin/P; Match between runs was used for identification transfer. Search results were filtered with 1% FDR at both protein and peptide levels. Significantly changed proteins were screened out according to the fold changes and p-values of the proteins. In this study, criteria with a $p$-value $< 0.05$ and fold change $> 2$ were taken for selection of nsp13-interacting proteins.

**Bioinformatic analysis of Gene Ontology (GO) and protein-protein interaction (PPI) network and data mining of druggable targets and host-directing drugs**

STRING database (Version 11.0) was used to evaluate the potential interactions of the identified cellular proteins and to group proteins into similar functional classes [23]. The protein-protein interaction (PPI) network was then visualized using Cytoscape software (Version 3.8) [24]. By STRING analysis, 830 interactions (edges) are represented in the protein-protein interaction network of the present study. Gene ontology (GO) analysis was used to determine the cellular component (CC), molecular function (MF) and biological process (BP) attributes of the identified nsp13-interacting cellular proteins by using the enricher function of clusterProfiler package in R with default parameters [25]. Significant GO terms (adjusted $p < 0.05$) were identified and further refined by the simplify method (cutoff = 0.6, by="*p*.adjust") to cut redundant gene sets.

Cellular nsp13-interacting proteins may positively or negatively regulate nsp13 function (hence bolstering or inhibiting viral infection), or in turn, may be affected by nsp13, leading to viral fitness or cellular dysfunction. Thus, the nsp13-cell interaction interface could be a promising target to intervene viral infection and pathogenesis. To explore potential druggable targets, the identified nsp13-interacting proteins were searched against DrugBank database [26]. Of the 172 cellular proteins interacting with nsp13 with high significance, 45 were returned with 95 targeting drugs (50 approved, 33 experimental, 10 investigational, and 2 nutraceutical). The cellular target-drugs network was then visualized with Cytoscape software [24].

**Immunofluorescence and confocal microscopy**

Cells were fixed with 4% paraformaldehyde in PBS for 10 min and permeabilized with 0.5% Triton X-100 in PBS. After blocking with 2.5% BSA and 2.5% normal goat serum in PBS, cells were incubated with primary antibodies overnight at 4 °C and then stained with secondary antibodies for 1 h at room temperature (RT). To visualize the nuclei, cells were treated with Hoechst 33258 (Beyotime) for 10 min at RT. Image acquisition and analyses were performed with a Nikon Ti confocal microscopy with the Volocity software (PerkinElmer).

### Statistical analysis

Statistical analyses were performed by GraphPad Prism 8 using unpaired two-tailed *t* test or Welch's *t* test. Data were expressed as mean ± SD, $n = 3$ or 4 biological replicates as indicated. $p < 0.05$ was considered statistically significant.

Supplementary Material

mjab068_Supplementary_Data

