# Interactome profiling reveals interaction of SARS-CoV-2 NSP13 with host factor STAT1 to suppress interferon signaling

**DOI:** 10.1093/jmcb/mjab068

**Published:** 2021-10-23

**Authors:** Kuan Feng, Yuan-Qin Min, Xiulian Sun, Fei Deng, Peiqing Li, Hualin Wang, Yun-Jia Ning

**Affiliations:** 1 State Key Laboratory of Virology, Wuhan Institute of Virology, Chinese Academy of Sciences, Wuhan 430071, China; 2 Department of Pediatric Emergency, Guangzhou Women and Children’s Medical Center, Guangzhou Medical University, Guangzhou 510623, China; 3 Center for Biosafety Mega-Science, Chinese Academy of Sciences, Wuhan 430071, China

**Dear Editor,**

The on-going coronavirus disease 2019 (COVID-19) pandemic caused by severe acute respiratory syndrome coronavirus 2 (SARS-CoV-2) has resulted in unprecedented medical and socioeconomic disruption globally. As of late September 2021, over 231 million confirmed cases have been reported worldwide. Although the viral pathogenesis remains largely unclear, impairment of interferon (IFN) responses likely contributes to disease progression and severity (Blanco-Melo et al., 2020; Meffre and Iwasaki, 2020; Min et al., 2021). Indeed, several viral proteins are potential regulators of the IFN system (Min et al., 2021). However, the underlying mechanisms employed by SARS-CoV-2 IFN antagonist candidates, such as nonstructural protein 13 (NSP13), still need to be determined. 

NSP13 is important in viral replication as a helicase unwinding duplex RNA and a 5′-triphosphatase likely involved in 5′-capping of viral mRNA (Min et al., 2021). We observed robust inhibition of type I IFN signaling by SARS-CoV-2 NSP13 through reporter gene assays (Figure 1A), consistent with previous observations (Lei et al., 2020; Xia et al., 2020; Yuen et al., 2020), highlighting the versatile activities of NSP13 in viral infection. Furthermore, NSP13 inhibition of type I IFN signaling was confirmed by quantitative polymerase chain reaction analyses of the induction of antiviral IFN-stimulated genes (ISGs) (Supplementary Figure S1A). Although Gordon et al. (2020) recently reported possible host molecules interacting with SARS-CoV-2 proteins, none of the 40 cellular proteins identified with NSP13 as bait are involved in IFN-triggered signaling pathways. Additionally, time-sensitive preparation of the manuscript precluded visual results (immunoblotting or silver staining) demonstrating effective precipitation of baits and cellular proteins before mass spectrometry (MS) (Gordon et al., 2020). Thus, further exploration of the viral interactome is worthwhile.

**Figure 1:**
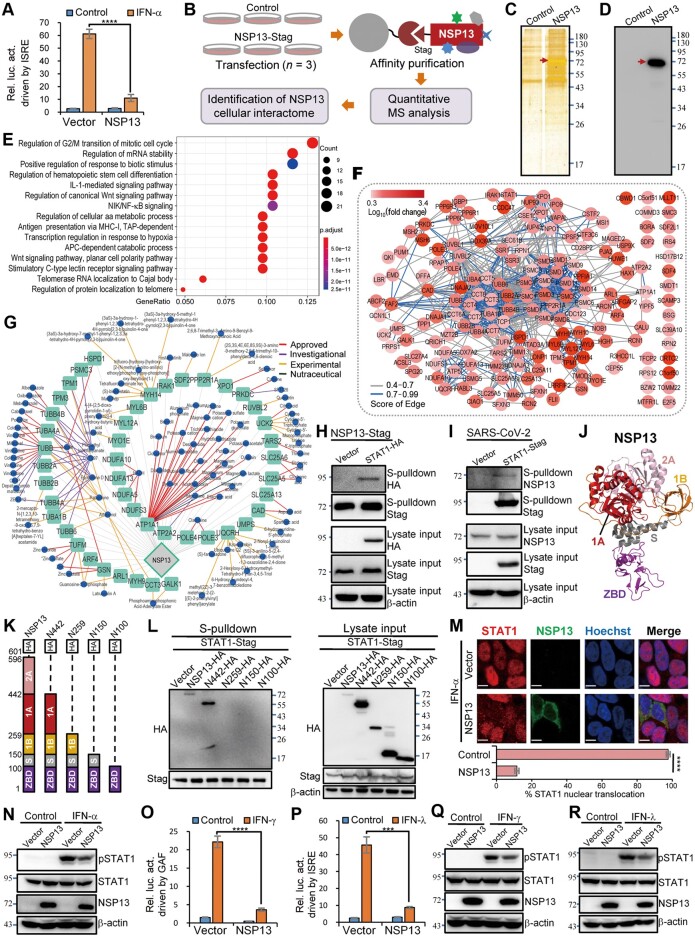
A new interactome of SARS-CoV-2 NSP13 reveals STAT1 as the cellular interaction target for NSP13 antagonism of IFN signaling cascades. (**A**) NSP13 suppresses type I IFN-triggered signaling. Dual-luciferase reporter (DLR) assays were conducted as described in Supplementary Materials and methods. Relative luciferase activity (Rel. luc. act.) is shown. (**B**) Workflow for identifying cellular proteins interacting with NSP13. (**C** and **D**) Silver-stained sodium dodecyl sulfate−polyacrylamide gel and immunoblot of control or NSP13 pulldown products. Red arrows indicate efficient precipitation of NSP13. (**E** and **F**) Biological process enrichment (**E**) and STRING analysis (**F**) for NSP13-interacting proteins (fold change >2, *P *<* *0.05). The top 15 enriched gene ontologies are shown (**E**). (**G**) Drug–cellular target network. (**H**) Validation of NSP13–STAT1 interaction using S-pulldown (NSP13 as bait) and immunoblotting with HEK293T cells. (**I**) Reciprocal S-pulldown assay using HEK293-ACE2 cells infected with SARS-CoV-2, further corroborating NSP13–STAT1 interaction. S-pulldown assay was performed at 24 h postinfection. (**J**) 3D structure of NSP13 (PDB: 6ZSL). (**K**) Linear representation of domain organization in full-length or C-terminal-truncated NSP13 (fused with HA tag). (**L**) S-pulldown and immunoblotting showing interactions of truncated NSP13 proteins with STAT1 in HEK293T cells. (**M**) STAT1 nuclear translocation detected using confocal microscopy. At 24 h post-transfection, HEK293 cells transfected with control or HA-tagged NSP13 expression plasmids were treated with IFN-α (2000 U/ml) for 30 min before immunofluorescence assay with anti-HA or anti-STAT1 antibodies; >150 cells for each group were then scored for STAT1 nuclear translocation. Scale bar, 7 µm. (**N**) HEK293T cells transfected with vector or S-tagged NSP13 expression plasmid were left untreated or treated with IFN-α (2000 U/ml) for 30 min, followed by immunoblotting. (**O** and **P**) NSP13 inhibits signal transduction driven by type II (IFN-γ; **O**) and type III (IFN-λ; **P**) IFNs. DLR assays for IFN-γ and IFN-λ signaling were performed. (**Q** and **R**) Cells were treated as in **N**, except for replacement of IFN-α by IFN-γ (100 ng/ml) or IFN-λ (200 ng/ml). Data in histograms are mean ± SD, *n *=* *4 (**A**, **O**, and **P**) or 3 (**M**). ****P <* *0.0001, ***P <* *0.001. Numbers beside gels or blots indicate molecular mass (kDa).

To elucidate the mechanism of NSP13-mediated IFN signaling antagonism and further decipher the potential cellular interaction landscape of such an important viral protein, we exploited a high-affinity purification assay (S-pulldown) coupled with quantitative MS to identify the cellular interactome of NSP13 (Figure 1B). S-tagged NSP13 expressed in cells was efficiently precipitated, and bands of cellular proteins co-precipitating with NSP13 were also evident (Figure 1C and D). MS analysis identified 172 cellular proteins as potential NSP13-interacting host factors with high confidence (Supplementary Table S1 and Figure S2A); 171 were newly identified in this study (C1orf50 was also reported by Gordon et al., 2020). These proteins might affect NSP13 function or be affected by NSP13. We next validated the interactions of NSP13 with a series of representative host proteins through immunoblotting (Supplementary Figure S3), further supporting the new interactome. Gene ontology and protein–protein interaction network analyses showed that these host proteins were enriched in several important biological processes and some specific molecular functions and cellular components (Figure 1E and F; Supplementary Figure S2D and E), highlighting novel and possibly significant aspects of SARS-CoV-2 biology. For instance, enrichment of NSP13-interacting proteins in regulation of mRNA stability might be linked to the role of NSP13 in viral mRNA capping. Interactions of NSP13 with host proteins involved in IL-1 signaling and MHC-I antigen presentation may modulate these pivotal inflammatory and immune responses during SARS-CoV-2 infection. Intriguingly, the NSP13 interactome was enriched in proteins associated with transcriptional regulation in response to hypoxia (a pivotal and concerning pathophysiological feature of COVID-19 pneumonia; Kashani, 2020), indicating novel potential roles of NSP13 in COVID-19 pathogenesis that merit future exploration. Furthermore, we identified 45 pharmacological cellular targets along with 95 corresponding drugs by drug database mining with the interactome (Figure 1G;  Supplementary Table S2), revealing potentially valuable clues for future design of a NSP13–host interface-directed intervention strategy.

Importantly, an essential component of the IFN-triggered signaling pathway, STAT1 (Najjar and Fagard, 2010), was identified as a potential NSP13-interacting protein (Supplementary Table S1 and Figure S2A–C). Given the central role of STAT1 in IFN signaling cascades (Najjar and Fagard, 2010), we speculated that STAT1 might be the cellular interaction target of NSP13 for IFN signaling counteraction. Immunoblott ing indicated that STAT1 indeed co-precipitated with NSP13 (Figure 1H). Moreover, reciprocal interaction analysis in the context of SARS-CoV-2 infection confirmed co-precipitation of NSP13 with STAT1 (Figure 1I). The NSP13–STAT1 interaction was further demonstrated to be independent of RNA or DNA using a nuclease treatment assay (Mo et al., 2020) before protein-interaction detection (Supplementary Figure S4). Then, we constructed a series of C-terminal truncations based on NSP13 3D structure to map the interaction domain (Figure 1J and K). Interestingly, truncated NSP13 with the 2A domain deleted (N442) retained interaction with STAT1, whereas removal of the 1A domain (N259) or further deletions (N150 or N100) abolished NSP13–STAT1 interaction (Figure 1L), indicating a necessary role of 1A domain (Figure 1J) in NSP13 targeting STAT1. Furthermore, immunofluorescence assays demonstrated that NSP13 expression blocks nuclear accumulation of STAT1 induced by IFN-α (Figure 1M). Interestingly, NSP13 mutant N259 failed to block STAT1 nuclear translocation, while mutant N442 retained inhibitory ability against STAT1 nuclear accumulation (Supplementary Figure S5), in line with their interactions with STAT1 (Figure 1L). IFN-α-triggered STAT1 phosphorylation, a prerequisite for STAT1 activation and efficient nuclear translocation, was noticeably reduced by NSP13 (Figure 1N). Moreover, inhibition of IFN-α-stimulated STAT1 activation by NSP13 was comparable to or stronger than that by SOCS1 (Supplementary Figure S6A), a well-known cellular negative regulator of Janus kinase (Najjar and Fagard, 2010). These consistent observations corroborate that NSP13 suppresses type I IFN signaling by interacting with STAT1 and hence impairing the host protein’s actions. As STAT1 is an indispensable signaling molecule shared by all type I, type II, and type III IFN signaling cascades, we considered that NSP13 might antagonize type II and type III IFN signaling as well. Indeed, NSP13 expression significantly inhibited both IFN-γ- and IFN-λ-induced reporter activation (Figure 1O and P) and antiviral ISG expression (Supplementary Figure S1B and C). Furthermore, STAT1 phosphorylation triggered by type II and type III IFNs was also suppressed by NSP13 (Figure 1Q and R; Supplementary Figure S6B and C). Together, these data suggest that NSP13 targets STAT1 through interaction requiring the NSP13 1A domain, thus interfering with STAT1 actions. The interference ultimately inhibits type I, type II, and type III IFN signaling cascades that orchestrate complex host immune responses upon viral infection.

In summary, we uncovered a new interactome of the critical viral protein NSP13, revealing important aspects of SARS-CoV-2 biology as well as potential druggable targets and targeting drugs at the interface of virus–host interactions. We further demonstrated that nucleic acid-independent interaction of NSP13 with STAT1 involves NSP13 domain 1A and results in inhibition of type I, type II, and type III IFN-elicited STAT1 actions. Given the IFN-antagonistic and hence potential virulence-associated activities of NSP13, future studies of specific NSP13 mutant viruses (e.g. those constructed by reverse genetics) will further elucidate the biological significance of NSP13 targeting of STAT1 or other host factors and help inform the design of attenuated vaccines. Our findings unravel the cellular interaction target and mechanism for NSP13 blockade of IFN signaling and provide new insights into virus–host interactions from molecular and proteomic perspectives, which benefit not only better understanding of SARS-CoV-2 infection but also further development of antiviral therapies.

*[Supplementary material is available at Journal of Molecular Cell Biology online. We thank Dr Hong-Bing Shu (Wuhan University) for providing the reporter plasmids, Dr Xinwen Chen (Wuhan Institute of Virology) for the viral vector packaging plasmids, the National Virus Resource Center (Wuhan Institute of Virology) for virus resources, and the Core Facility and Technical Support of Wuhan Institute of Virology for technical assistance. We are also grateful to the administration team of the National Biosafety Laboratory, Wuhan, Chinese Academy of Sciences. This work was supported by grants from the National Natural Science Foundation of China (31870162 and 82161138003), the National Key Research and Development Program of China (2018YFA0507202), and the Youth Innovation Promotion Association of Chinese Academy of Sciences.]*

## Supplementary Material

mjab068_Supplementary_Data
