# Supplementary material for: Interactome profiling reveals interaction of SARS-CoV-2 NSP13 with host factor STAT1 to suppress interferon signaling
Source: J Mol Cell Biol. 2021 Oct 23;13(10):760–2. doi: 10.1093/jmcb/mjab068 (PMC8574307; doi:10.1093/jmcb/mjab068)
Supplement: mjab068_Supplementary_Data [file mjab068_supplementary_data.pdf]

Supplementary materials and methods

Cells and virus

HEK293 (ATCC, CRL-1573) and Vero E6 cells (ATCC, CRL-1586) were maintained in Eagle's minimum essential medium supplemented with 10% fetal bovine serum (FBS) at 37 °C in a humidified atmosphere with 5% CO_2. HEK293T (ATCC, CRL-11268) cells were cultured in Dulbecco's modified Eagle's medium. HEK293-ACE2 cell line was generated by transduction of lentiviral vector expressing ACE2 and subsequent monoclonal screening as described below. SARS-CoV-2 (nCoV-2019BetaCoV/Wuhan/WIV04/2019) was isolated from the clinical sample [1]. The propagation and titer determination of SARS-CoV-2 were performed in Vero E6 cells in a biosafety level-3 (BSL-3) laboratory of National Biosafety Laboratory, Wuhan, Chinese Academy of Sciences [1].

Plasmids

Open reading frame (ORF) encoding NSP13 was amplified by RT-PCR from SARS-CoV-2 genomic RNA and cloned into the pCAGGSP7 vector without or with HA or S tag (Stag) [2-4]. Truncated NSP13 and SOCS1 expression plasmids were constructed into pCAGGSP7 vector with C-terminal HA tag by standard molecular biology methods. S or HA-tagged STAT1 expression plasmids were constructed as previously described [3, 5-7]. Human angiotensin I-converting enzyme-2 (ACE2) expression plasmid (pLVX-IRES-PURO-ACE2) was generated by cloning the ORF sequence of ACE2 into lentiviral vector pLVX-IRES-PURO. Luciferase reporter plasmids for type I, II, or III IFN-responsive promoters and the *Renilla* luciferase control plasmid (pRL-TK) were kindly provided by Prof. Hong-Bing Shu (Wuhan University) and were described previously [3, 6, 8-11].

Antibodies and Reagents

Primary antibodies include mouse antibody to HA tag (Sigma-Aldrich, 05–904) and rabbit antibodies to S tag (Abcam, ab18588), β-actin (ABclonal, AC026), NSP13

(AtaGenix, ATP238), STAT1 (Abcam, ab109320), pSTAT1 (Cell Signaling Technology, 9167S), IRAK1 (proteintech, 10478-2-AP), HAX1 (proteintech, 11266-1-AP), DNAJA2 (ABclonal, A7059), HSPD1 (ABclonal, A0564), CCT4 (ABclonal, A6548), CCT8 (ABclonal, A4449), and TCP1 (ABclonal, A1950). For Immunofluorescence assay (IFA), secondary antibodies include Alexa Fluor 647-conjugated goat anti-rabbit IgG (H+L) (Abcam, ab150079) and Alexa Fluor 488-conjugated goat anti-mouse IgG (H+L) (Abcam, ab150113). For WB analysis, secondary antibodies include horseradish peroxidase (HRP)-conjugated goat anti-mouse or anti-rabbit IgG antibodies (Abcam, ab6789 and ab6721). Recombinant human IFN-α (300-02AA), IFN-γ (300-02), and IFN-λ (300-02K) were all purchased from Peprotech.

Real-time qPCR

Total RNA was extracted from the treated HEK293T cells with TRIzol reagent (NZK biotech, China). Reverse transcription of RNA was performed with a cDNA reverse transcription kit (TaKaRa, RR047A), followed by real-time quantitative PCR with an SYBR Green real-time PCR kit (TaKaRa, RR820A). The relative mRNA levels of the indicated genes were calculated using the $2^{-\Delta\Delta CT}$ method with *GAPDH* mRNA levels as an internal control. Primers used have been described in previous studies [12-15].

Dual-luciferase reporter (DLR) gene assay

HEK293T cells cultured in 96-well plates were cotransfected with 80 ng NSP13 expression plasmid or the control vector, together with 20 ng ISRE (for type I and III IFN signaling analyses) or GAF reporter plasmid (for type II IFN signaling analysis) and 0.5 ng pRL-TK, using lipofectamine 3000 (Invitrogen) by following the manufacturer’s instructions. At 24 h posttransfection, cells were mock stimulated or stimulated with IFN-α (type I IFN; 2000 u/ml), IFN-γ (type II IFN; 100 ng/ml), or IFN-λ (type III IFN; 200 ng/ml) for 16 h before measurements of luciferase activities with a DLR assay kit (Promega, E2940). The relative luciferase activities (Rel. Luc. Act) were exhibited by normalizing firefly luciferase activity to *Renilla* luciferase

activity.

Generation of HEK293-ACE2 cell line

HEK293T cells were co-transfected with the human ACE2 expression plasmid pLVX-IRES-PURO-ACE2 and packaging plasmids provided by Prof. Xinwen Chen (Wuhan Institute of Virology) for 48 h [14, 16]. Packaged lentiviral particles were then harvested from culture medium and used for transduction of HEK293 cells, followed by 3-day selection with 2 μg/mL puromycin. Then, cells were diluted and seeded into 96-well plates for further screening of single colony stably over-expressing ACE2.

Protein interaction, silver staining, and WB analyses

S-pulldown assays utilizing the high-specific and strong affinity of S tag with S-protein coupled on agarose beads were performed as described previously to analyze protein interactions [3, 5, 14, 17-19]. In cotransfection assays before S-pulldown, HEK293T cells were transfected with the S-tagged protein expression plasmids together with the plasmids encoding other indicated proteins for 36 h. For protein interaction analysis in the contexts of infection combined with transfection, HEK293-ACE2 cells were firstly transfected with the plasmid encoding STAT1-Stag or the control vector and at 12 h posttransfection, the cells were infected with SARS-CoV-2 (MOI = 1) for 24 h. Then, the transfected or SARS-CoV-2-infected cells expressing S-tagged proteins were lysed with the immunoprecipitation lysis buffer (Beyotime) and after centrifugation, supernatants of cell lysates were mixed with S-protein agarose (Millipore) by rotating incubation. The beads were next washed with the lysis buffer and phosphate-buffered saline (PBS) successively, followed by silver staining, WB, or quantitative MS analyses of the bound protein samples. For silver staining analysis, protein samples were eluted with 1×SDS sample buffer by boiling for 10min and subjected to 10% SDS polyacrylamide gel electrophoresis (SDS-PAGE). Then, the gel was fixed, sensitized, and stained by Pierce Silver Stain Kit (Thermo Fisher Scientific) according to the manufacturer's

instructions. For WB analysis, protein samples separated by SDS-PAGE were transferred onto polyvinylidene difluoride (PVDF) membranes. After blocking with 5% BSA in Tris-buffered saline-Tween 20 (TBS-T), the membranes were incubated successively with primary antibodies and HRP-conjugated secondary antibodies. Protein bands were detected with an enhanced chemiluminescence kit (Thermo Fisher Scientific). In nuclease treatment assays, cell lysates (supplemented with 2 mM MgCl2) were divided into halves and then subjected to excessive treatment with Benzonase (100 U, Millipore, Cat#70746) or left untreated at 4˚C for 4 h before S-pulldown as described previously [14].

Quantitative MS analysis

For MS quantification analysis, bead-bound proteins (in total of 6 samples from three biological replicates for both NSP13 pulldown and control groups) were denatured, reduced, alkylated, and eluted by one-step treatment in the reaction buffer (1% SDC/100 mM Tris-HCl, pH 8.5/10 mM TCEP/40 mM CAA) at 95 °C for 10 min. The eluates were diluted with equal volume of H_2O and then subjected to trypsin digestion at 37 °C overnight. Obtained peptides were desalted for liquid chromatography-tandem mass spectrometry (LC-MS/MS) analysis using a Q Exactive HF-X system coupled with Easy-nLC 1200 (Thermo Fisher Scientific) [20]. MS raw tandem spectra were analyzed with MaxQuant (V1.6.6) (AB SCIEX) using the Andromeda database search algorithm [21]. Spectra files were searched against the Human Unified Protein Database (UniProt) [22]. The main parameters were as follows: LFQ mode was checked for quantification; Variable modifications, Oxidation (M) & Acetyl (Protein N-term); Fixed modifications, Carbamidomethyl (C); Digestion, Trypsin/P; Match between runs was used for identification transfer. Search results were filtered with 1% FDR at both protein and peptide levels. Significantly changed proteins were screened out according to the fold changes and p-values of the proteins. In this study, criteria with a p-value < 0.05 and fold change > 2 were taken for selection of NSP13-interacting proteins.

Bioinformatic analysis of Gene Ontology (GO) and protein-protein interaction (PPI) network and data mining of druggable targets and host-directing drugs

STRING database (Version 11.0) was used to evaluate the potential interactions of the identified cellular proteins and to group proteins into similar functional classes [23]. The protein-protein interaction (PPI) network was then visualized using Cytoscape software (Version 3.8) [24]. By STRING analysis, 830 interactions (edges) are represented in the protein-protein interaction network of the present study. Gene ontology (GO) analysis was used to determine the cellular component (CC), molecular function (MF) and biological process (BP) attributes of the identified NSP13-interacting cellular proteins by using the enricher function of clusterProfiler package in R with default parameters [25]. Significant GO terms (adjusted $p < 0.05$) were identified and further refined by the simplify method (cutoff = 0.6, by="*p*.adjust") to cut redundant gene sets.

Cellular NSP13-interacting proteins may positively or negatively regulate NSP13 function (hence bolstering or inhibiting viral infection), or in turn, may be affected by NSP13, leading to viral fitness or cellular dysfunction. Thus, the NSP13-cell interaction interface could be a promising target to intervene viral infection and pathogenesis. To explore potential druggable targets, the identified NSP13-interacting proteins were searched against DrugBank database [26]. Of the 172 cellular proteins interacting with NSP13 with high significance, 45 were returned with 95 targeting drugs (50 approved, 33 experimental, 10 investigational, and 2 nutraceutical). The cellular target-drugs network was then visualized with Cytoscape software [24].

Immunofluorescence and confocal microscopy

Cells were fixed with 4% paraformaldehyde in PBS for 10 min and permeabilized with 0.5% Triton X-100 in PBS. After blocking with 2.5% BSA and 2.5% normal goat serum in PBS, cells were incubated with primary antibodies overnight at 4 °C and then stained with secondary antibodies for 1 h at room temperature (RT). To visualize the nuclei, cells were treated with Hoechst 33258 (Beyotime) for 10 min at RT. Image acquisition and analyses were performed with a Nikon Ti confocal microscopy with

the Volocity software (PerkinElmer).

Statistical analysis

Statistical analyses were performed by GraphPad Prism 8 using unpaired two-tailed *t* test or Welch's *t* test. Data were expressed as mean ± SD, $n = 3$ or 4 biological replicates as indicated. $p < 0.05$ was considered statistically significant.

References

1. **Zhou, P., X.-L. Yang, X.-G. Wang, B. Hu, L. Zhang, W. Zhang, H.-R. Si, Y. Zhu, B. Li, C.-L. Huang, H.-D. Chen, J. Chen, Y. Luo, H. Guo, R.-D. Jiang, M.-Q. Liu, Y. Chen, X.-R. Shen, X. Wang, X.-S. Zheng, K. Zhao, Q.-J. Chen, F. Deng, L.-L. Liu, B. Yan, F.-X. Zhan, Y.-Y. Wang, G.-F. Xiao, and Z.-L. Shi.** 2020. A pneumonia outbreak associated with a new coronavirus of probable bat origin. Nature.
2. **Ning, Y.-J., M. Wang, M. Deng, S. Shen, W. Liu, W.-C. Cao, F. Deng, Y.-Y. Wang, Z. Hu, and H. Wang.** 2014. Viral suppression of innate immunity via spatial isolation of TBK1/IKKepsilon from mitochondrial antiviral platform. J. Mol. Cell. Biol. **6:**324-337.
3. **Ning, Y.-J., K. Feng, Y.-Q. Min, W.-C. Cao, M. Wang, F. Deng, Z. Hu, and H. Wang.** 2015. Disruption of type I interferon signaling by the nonstructural protein of severe fever with thrombocytopenia syndrome virus via the hijacking of STAT2 and STAT1 into inclusion bodies. J. Virol. **89:**4227-4236.
4. **Ning, Y.-J., Z. Kang, J. Xing, Y.-Q. Min, D. Liu, K. Feng, M. Wang, F. Deng, Y. Zhou, Z. Hu, and H. Wang.** 2018. Ebola virus mucin-like glycoprotein (Emuc) induces remarkable acute inflammation and tissue injury: evidence for Emuc pathogenicity in vivo. Protein & cell **9:**389-393.
5. **Feng, K., F. Deng, Z. Hu, H. Wang, and Y.-J. Ning.** 2019. Heartland virus antagonizes type I and III interferon antiviral signaling by inhibiting phosphorylation and nuclear translocation of STAT2 and STAT1. The Journal of biological chemistry **294:**9503-9517.
6. **Ning, Y.-J., Q. Mo, K. Feng, Y.-Q. Min, M. Li, D. Hou, C. Peng, X. Zheng, F. Deng, Z. Hu, and H. Wang.** 2019. Interferon-gamma-Directed Inhibition of a Novel High-Pathogenic Phlebovirus and Viral Antagonism of the Antiviral Signaling by Targeting STAT1. Front. Immunol. **10:**1182.
7. **Min, Y.-Q., C. Shi, T. Yao, K. Feng, Q. Mo, F. Deng, H. Wang, and Y.-J. Ning.** 2020. The Nonstructural Protein of Guertu Virus Disrupts Host Defenses by Blocking Antiviral Interferon Induction and Action. ACS Infect Dis **6:**857-870.
8. **Ran, Y., J. Zhang, L.-L. Liu, Z.-Y. Pan, Y. Nie, H.-Y. Zhang, and Y.-Y. Wang.** 2016. Autoubiquitination of TRIM26 links TBK1 to NEMO in RLR-mediated innate antiviral immune response. J Mol Cell Biol **8:**31-43.
9. **Li, Y., R. Chen, Q. Zhou, Z. Xu, C. Li, S. Wang, A. Mao, X. Zhang, W. He, and H.-B. Shu.** 2012. LSm14A is a processing body-associated sensor of viral nucleic acids that initiates cellular antiviral response in the early phase of viral infection. Proc Natl Acad Sci U S A **109:**11770-11775.

10. **Wang, Y. Y., L. J. Liu, B. Zhong, T. T. Liu, Y. Li, Y. Yang, Y. Ran, S. Li, P. Tien, and H. B. Shu.** 2010. WDR5 is essential for assembly of the VISA-associated signaling complex and virus-triggered IRF3 and NF-kappaB activation. Proc Natl Acad Sci U S A **107:**815-820.

11. **Zhong, B., Y. Yang, S. Li, Y. Y. Wang, Y. Li, F. Diao, C. Lei, X. He, L. Zhang, P. Tien, and H. B. Shu.** 2008. The adaptor protein MITA links virus-sensing receptors to IRF3 transcription factor activation. Immunity **29:**538-550.

12. **Ning, Y. J., Q. Mo, K. Feng, Y. Q. Min, M. Y. Li, D. H. Hou, C. Peng, X. Zheng, F. Deng, Z. H. Hu, and H. L. Wang.** 2019. Interferon-gamma-Directed Inhibition of a Novel High-Pathogenic Phlebovirus and Viral Antagonism of the Antiviral Signaling by Targeting STAT1. Front Immunol **10**.

13. **Ning, Y. J., K. Feng, Y. Q. Min, W. C. Cao, M. Wang, F. Deng, Z. Hu, and H. Wang.** 2015. Disruption of type I interferon signaling by the nonstructural protein of severe fever with thrombocytopenia syndrome virus via the hijacking of STAT2 and STAT1 into inclusion bodies. J Virol **89:**4227-4236.

14. **Mo, Q., Z. Xu, F. Deng, H. Wang, and Y.-J. Ning.** 2020. Host restriction of emerging high-pathogenic bunyaviruses via MOV10 by targeting viral nucleoprotein and blocking ribonucleoprotein assembly. PLoS Pathog **16:**e1009129.

15. **Ning, Y. J., K. Feng, Y. Q. Min, F. Deng, Z. H. Hu, and H. L. Wang.** 2017. Heartland virus NSs protein disrupts host defenses by blocking the TBK1 kinase-IRF3 transcription factor interaction and signaling required for interferon induction. J Biol Chem **292:**16722-16733.

16. **Min, Y.-Q., Y.-J. Ning, H. Wang, and F. Deng.** 2020. A RIG-I-like receptor directs antiviral responses to a bunyavirus and is antagonized by virus-induced blockade of TRIM25-mediated ubiquitination. The Journal of biological chemistry **295:**9691-9711.

17. **Hall, E. H., J. L. Balsbaugh, K. L. Rose, J. Shabanowitz, D. F. Hunt, and D. L. Brautigan.** 2010. Comprehensive analysis of phosphorylation sites in Tensin1 reveals regulation by p38MAPK. Mol Cell Proteomics **9:**2853-2863.

18. **Ning, Y.-J., K. Feng, Y.-Q. Min, F. Deng, Z. Hu, and H. Wang.** 2017. Heartland virus NSs protein disrupts host defenses by blocking the TBK1 kinase-IRF3 transcription factor interaction and signaling required for interferon induction. The Journal of biological chemistry **292:**16722-16733.

19. **Belgnaoui, S. M., S. Paz, S. Samuel, M.-L. Goulet, Q. Sun, M. Kikkert, K. Iwai, I. Dikic, J. Hiscott, and R. Lin.** 2012. Linear ubiquitination of NEMO negatively regulates the interferon antiviral response through disruption of the MAVS-TRAF3 complex. Cell Host Microbe **12:**211-222.

20. **Kelstrup, C. D., D. B. Bekker-Jensen, T. N. Arrey, A. Hogrebe, A. Harder, and J. V. Olsen.** 2018. Performance Evaluation of the Q Exactive HF-X for Shotgun Proteomics. J Proteome Res **17:**727-738.

21. **Cox, J., M. Y. Hein, C. A. Luber, I. Paron, N. Nagaraj, and M. Mann.** 2014. Accurate proteome-wide label-free quantification by delayed normalization and maximal peptide ratio extraction, termed MaxLFQ. Mol Cell Proteomics **13:**2513-2526.

22. **TheUniProtConsortium.** 2017. UniProt: the universal protein knowledgebase. Nucleic Acids Res **45:**D158-D169.

23. **Szklarczyk, D., A. L. Gable, D. Lyon, A. Junge, S. Wyder, J. Huerta-Cepas, M. Simonovic, N. T. Doncheva, J. H. Morris, P. Bork, L. J. Jensen, and C. v. Mering.** 2019. STRING v11:

protein-protein association networks with increased coverage, supporting functional discovery in genome-wide experimental datasets. Nucleic Acids Res **47:**D607-D613.

24. **Shannon, P., A. Markiel, O. Ozier, N. S. Baliga, J. T. Wang, D. Ramage, N. Amin, B. Schwikowski, and T. Ideker.** 2003. Cytoscape: a software environment for integrated models of biomolecular interaction networks. Genome Res **13:**2498-2504.
25. **Yu, G., L.-G. Wang, Y. Han, and Q.-Y. He.** 2012. clusterProfiler: an R package for comparing biological themes among gene clusters. Omics **16:**284-287.
26. **Wishart, D. S., Y. D. Feunang, A. C. Guo, E. J. Lo, A. Marcu, J. R. Grant, T. Sajed, D. Johnson, C. Li, Z. Sayeeda, N. Assempour, I. Iynkkaran, Y. Liu, A. Maciejewski, N. Gale, A. Wilson, L. Chin, R. Cummings, D. Le, A. Pon, C. Knox, and M. Wilson.** 2018. DrugBank 5.0: a major update to the DrugBank database for 2018. Nucleic Acids Res **46:**D1074-D1082.
