# Supplementary material for: Interactome profiling reveals interaction of SARS-CoV-2 NSP13 with host factor STAT1 to suppress interferon signaling
Source: J Mol Cell Biol. 2021 Oct 23:mjab068. doi: 10.1093/jmcb/mjab068 (PMC8574307; doi:10.1093/jmcb/mjab068)
Supplement: Supplementary file 1 [file mjab068_supplementary_data.pdf]

Supplementary materials and methods

Cells and virus

HEK293 (ATCC, CRL-1573) and Vero E6 cells (ATCC, CRL-1586) were maintained in Eagle's minimum essential medium supplemented with 10% fetal bovine serum (FBS) at 37 °C in a humidified atmosphere with 5% CO_2. HEK293T (ATCC, CRL-11268) cells were cultured in Dulbecco's modified Eagle's medium. HEK293-ACE2 cell line was generated by transduction of lentiviral vector expressing ACE2 and subsequent monoclonal screening as described below. SARS-CoV-2 (nCoV-2019BetaCoV/Wuhan/WIV04/2019) was isolated from the clinical sample [1]. The propagation and titer determination of SARS-CoV-2 were performed in Vero E6 cells in a biosafety level-3 (BSL-3) laboratory of National Biosafety Laboratory, Wuhan, Chinese Academy of Sciences [1].

Plasmids

Open reading frame (ORF) encoding nsp13 was amplified by RT-PCR from SARS-CoV-2 genomic RNA and cloned into the pCAGGSP7 vector without or with HA or S tag (Stag) [2-4]. Truncated nsp13 and SOCS1 expression plasmids were constructed into pCAGGSP7 vector with C-terminal HA tag by standard molecular biology methods. S or HA-tagged STAT1 expression plasmids were constructed as previously described [3, 5-7]. Human angiotensin I-converting enzyme-2 (ACE2) expression plasmid (pLVX-IRES-PURO-ACE2) was generated by cloning the ORF sequence of ACE2 into lentiviral vector pLVX-IRES-PURO. Luciferase reporter plasmids for type I, II, or III IFN-responsive promoters and the *Renilla* luciferase control plasmid (pRL-TK) were kindly provided by Prof. Hong-Bing Shu (Wuhan University) and were described previously [3, 6, 8-11].

Antibodies and Reagents

Primary antibodies include mouse antibody to HA tag (Sigma-Aldrich, 05–904) and rabbit antibodies to S tag (Abcam, ab18588), β-actin (ABclonal, AC026), nsp13

(AtaGenix, ATP238), STAT1 (Abcam, ab109320), pSTAT1 (Cell Signaling Technology, 9167S), IRAK1 (proteintech, 10478-2-AP), HAX1 (proteintech, 11266-1-AP), DNAJA2 (ABclonal, A7059), HSPD1 (ABclonal, A0564), CCT4 (ABclonal, A6548), CCT8 (ABclonal, A4449), and TCP1 (ABclonal, A1950). For Immunofluorescence assay (IFA), secondary antibodies include Alexa Fluor 647-conjugated goat anti-rabbit IgG (H+L) (Abcam, ab150079) and Alexa Fluor 488-conjugated goat anti-mouse IgG (H+L) (Abcam, ab150113). For WB analysis, secondary antibodies include horseradish peroxidase (HRP)-conjugated goat anti-mouse or anti-rabbit IgG antibodies (Abcam, ab6789 and ab6721). Recombinant human IFN-α (300-02AA), IFN-γ (300-02), and IFN-λ (300-02K) were all purchased from Peprotech.

Real-time qPCR

Total RNA was extracted from the treated HEK293T cells with TRIzol reagent (NZK biotech, China). Reverse transcription of RNA was performed with a cDNA reverse transcription kit (TaKaRa, RR047A), followed by real-time quantitative PCR with an SYBR Green real-time PCR kit (TaKaRa, RR820A). The relative mRNA levels of the indicated genes were calculated using the $2^{-\Delta\Delta CT}$ method with *GAPDH* mRNA levels as an internal control. Primers used have been described in previous studies [12-15].

Dual-luciferase reporter (DLR) gene assay

HEK293T cells cultured in 96-well plates were cotransfected with 80 ng nsp13 expression plasmid or the control vector, together with 20 ng ISRE (for type I and III IFN signaling analyses) or GAF reporter plasmid (for type II IFN signaling analysis) and 0.5 ng pRL-TK, using lipofectamine 3000 (Invitrogen) by following the manufacturer's instructions. At 24 h posttransfection, cells were mock stimulated or stimulated with IFN-α (type I IFN; 2000 u/ml), IFN-γ (type II IFN; 100 ng/ml), or IFN-λ (type III IFN; 200 ng/ml) for 16 h before measurements of luciferase activities with a DLR assay kit (Promega, E2940). The relative luciferase activities (Rel. Luc. Act) were exhibited by normalizing firefly luciferase activity to *Renilla* luciferase

activity.

Generation of HEK293-ACE2 cell line

HEK293T cells were co-transfected with the human ACE2 expression plasmid pLVX-IRES-PURO-ACE2 and packaging plasmids provided by Prof. Xinwen Chen (Wuhan Institute of Virology) for 48 h [14, 16]. Packaged lentiviral particles were then harvested from culture medium and used for transduction of HEK293 cells, followed by 3-day selection with 2 μg/mL puromycin. Then, cells were diluted and seeded into 96-well plates for further screening of single colony stably over-expressing ACE2.

Protein interaction, silver staining, and WB analyses

S-pulldown assays utilizing the high-specific and strong affinity of S tag with S-protein coupled on agarose beads were performed as described previously to analyze protein interactions [3, 5, 14, 17-19]. In cotransfection assays before S-pulldown, HEK293T cells were transfected with the S-tagged protein expression plasmids together with the plasmids encoding other indicated proteins for 36 h. For protein interaction analysis in the contexts of infection combined with transfection, HEK293-ACE2 cells were firstly transfected with the plasmid encoding STAT1-Stag or the control vector and at 12 h posttransfection, the cells were infected with SARS-CoV-2 (MOI = 1) for 24 h. Then, the transfected or SARS-CoV-2-infected cells expressing S-tagged proteins were lysed with the immunoprecipitation lysis buffer (Beyotime) and after centrifugation, supernatants of cell lysates were mixed with S-protein agarose (Millipore) by rotating incubation. The beads were next washed with the lysis buffer and phosphate-buffered saline (PBS) successively, followed by silver staining, WB, or quantitative MS analyses of the bound protein samples. For silver staining analysis, protein samples were eluted with 1×SDS sample buffer by boiling for 10min and subjected to 10% SDS polyacrylamide gel electrophoresis (SDS-PAGE). Then, the gel was fixed, sensitized, and stained by Pierce Silver Stain Kit (Thermo Fisher Scientific) according to the manufacturer's

instructions. For WB analysis, protein samples separated by SDS-PAGE were transferred onto polyvinylidene difluoride (PVDF) membranes. After blocking with 5% BSA in Tris-buffered saline-Tween 20 (TBS-T), the membranes were incubated successively with primary antibodies and HRP-conjugated secondary antibodies. Protein bands were detected with an enhanced chemiluminescence kit (Thermo Fisher Scientific). In nuclease treatment assays, cell lysates (supplemented with 2 mM MgCl2) were divided into halves and then subjected to excessive treatment with Benzonase (100 U, Millipore, Cat#70746) or left untreated at 4˚C for 2 h before S-pulldown as described previously [14].

Quantitative MS analysis

For MS quantification analysis, bead-bound proteins (in total of 6 samples from three biological replicates for both nsp13 pulldown and control groups) were denatured, reduced, alkylated, and eluted by one-step treatment in the reaction buffer (1% SDC/100 mM Tris-HCl, pH 8.5/10 mM TCEP/40 mM CAA) at 95 °C for 10 min. The eluates were diluted with equal volume of H_2O and then subjected to trypsin digestion at 37 °C overnight. Obtained peptides were desalted for liquid chromatography-tandem mass spectrometry (LC-MS/MS) analysis using a Q Exactive HF-X system coupled with Easy-nLC 1200 (Thermo Fisher Scientific) [20]. MS raw tandem spectra were analyzed with MaxQuant (V1.6.6) (AB SCIEX) using the Andromeda database search algorithm [21]. Spectra files were searched against the Human Unified Protein Database (UniProt) [22]. The main parameters were as follows: LFQ mode was checked for quantification; Variable modifications, Oxidation (M) & Acetyl (Protein N-term); Fixed modifications, Carbamidomethyl (C); Digestion, Trypsin/P; Match between runs was used for identification transfer. Search results were filtered with 1% FDR at both protein and peptide levels. Significantly changed proteins were screened out according to the fold changes and p-values of the proteins. In this study, criteria with a p-value < 0.05 and fold change > 2 were taken for selection of nsp13-interacting proteins.

Bioinformatic analysis of Gene Ontology (GO) and protein-protein interaction (PPI) network and data mining of druggable targets and host-directing drugs

STRING database (Version 11.0) was used to evaluate the potential interactions of the identified cellular proteins and to group proteins into similar functional classes [23]. The protein-protein interaction (PPI) network was then visualized using Cytoscape software (Version 3.8) [24]. By STRING analysis, 830 interactions (edges) are represented in the protein-protein interaction network of the present study. Gene ontology (GO) analysis was used to determine the cellular component (CC), molecular function (MF) and biological process (BP) attributes of the identified nsp13-interacting cellular proteins by using the enricher function of clusterProfiler package in R with default parameters [25]. Significant GO terms (adjusted $p < 0.05$) were identified and further refined by the simplify method (cutoff = 0.6, by="*p*.adjust") to cut redundant gene sets.

Cellular nsp13-interacting proteins may positively or negatively regulate nsp13 function (hence bolstering or inhibiting viral infection), or in turn, may be affected by nsp13, leading to viral fitness or cellular dysfunction. Thus, the nsp13-cell interaction interface could be a promising target to intervene viral infection and pathogenesis. To explore potential druggable targets, the identified nsp13-interacting proteins were searched against DrugBank database [26]. Of the 172 cellular proteins interacting with nsp13 with high significance, 45 were returned with 95 targeting drugs (50 approved, 33 experimental, 10 investigational, and 2 nutraceutical). The cellular target-drugs network was then visualized with Cytoscape software [24].

Immunofluorescence and confocal microscopy

Cells were fixed with 4% paraformaldehyde in PBS for 10 min and permeabilized with 0.5% Triton X-100 in PBS. After blocking with 2.5% BSA and 2.5% normal goat serum in PBS, cells were incubated with primary antibodies overnight at 4 °C and then stained with secondary antibodies for 1 h at room temperature (RT). To visualize the nuclei, cells were treated with Hoechst 33258 (Beyotime) for 10 min at RT. Image acquisition and analyses were performed with a Nikon Ti confocal microscopy with

the Volocity software (PerkinElmer).

Statistical analysis

Statistical analyses were performed by GraphPad Prism 8 using unpaired two-tailed *t* test or Welch's *t* test. Data were expressed as mean ± SD, $n = 3$ or 4 biological replicates as indicated. $p < 0.05$ was considered statistically significant.

References

1. **Zhou, P., X.-L. Yang, X.-G. Wang, B. Hu, L. Zhang, W. Zhang, H.-R. Si, Y. Zhu, B. Li, C.-L. Huang, H.-D. Chen, J. Chen, Y. Luo, H. Guo, R.-D. Jiang, M.-Q. Liu, Y. Chen, X.-R. Shen, X. Wang, X.-S. Zheng, K. Zhao, Q.-J. Chen, F. Deng, L.-L. Liu, B. Yan, F.-X. Zhan, Y.-Y. Wang, G.-F. Xiao, and Z.-L. Shi.** 2020. A pneumonia outbreak associated with a new coronavirus of probable bat origin. Nature.
2. **Ning, Y.-J., M. Wang, M. Deng, S. Shen, W. Liu, W.-C. Cao, F. Deng, Y.-Y. Wang, Z. Hu, and H. Wang.** 2014. Viral suppression of innate immunity via spatial isolation of TBK1/IKKepsilon from mitochondrial antiviral platform. J. Mol. Cell. Biol. **6:**324-337.
3. **Ning, Y.-J., K. Feng, Y.-Q. Min, W.-C. Cao, M. Wang, F. Deng, Z. Hu, and H. Wang.** 2015. Disruption of type I interferon signaling by the nonstructural protein of severe fever with thrombocytopenia syndrome virus via the hijacking of STAT2 and STAT1 into inclusion bodies. J. Virol. **89:**4227-4236.
4. **Ning, Y.-J., Z. Kang, J. Xing, Y.-Q. Min, D. Liu, K. Feng, M. Wang, F. Deng, Y. Zhou, Z. Hu, and H. Wang.** 2018. Ebola virus mucin-like glycoprotein (Emuc) induces remarkable acute inflammation and tissue injury: evidence for Emuc pathogenicity in vivo. Protein & cell **9:**389-393.
5. **Feng, K., F. Deng, Z. Hu, H. Wang, and Y.-J. Ning.** 2019. Heartland virus antagonizes type I and III interferon antiviral signaling by inhibiting phosphorylation and nuclear translocation of STAT2 and STAT1. The Journal of biological chemistry **294:**9503-9517.
6. **Ning, Y.-J., Q. Mo, K. Feng, Y.-Q. Min, M. Li, D. Hou, C. Peng, X. Zheng, F. Deng, Z. Hu, and H. Wang.** 2019. Interferon-gamma-Directed Inhibition of a Novel High-Pathogenic Phlebovirus and Viral Antagonism of the Antiviral Signaling by Targeting STAT1. Front. Immunol. **10:**1182.
7. **Min, Y.-Q., C. Shi, T. Yao, K. Feng, Q. Mo, F. Deng, H. Wang, and Y.-J. Ning.** 2020. The Nonstructural Protein of Guertu Virus Disrupts Host Defenses by Blocking Antiviral Interferon Induction and Action. ACS Infect Dis **6:**857-870.
8. **Ran, Y., J. Zhang, L.-L. Liu, Z.-Y. Pan, Y. Nie, H.-Y. Zhang, and Y.-Y. Wang.** 2016. Autoubiquitination of TRIM26 links TBK1 to NEMO in RLR-mediated innate antiviral immune response. J Mol Cell Biol **8:**31-43.
9. **Li, Y., R. Chen, Q. Zhou, Z. Xu, C. Li, S. Wang, A. Mao, X. Zhang, W. He, and H.-B. Shu.** 2012. LSm14A is a processing body-associated sensor of viral nucleic acids that initiates cellular antiviral response in the early phase of viral infection. Proc Natl Acad Sci U S A **109:**11770-11775.

10. **Wang, Y. Y., L. J. Liu, B. Zhong, T. T. Liu, Y. Li, Y. Yang, Y. Ran, S. Li, P. Tien, and H. B. Shu.** 2010. WDR5 is essential for assembly of the VISA-associated signaling complex and virus-triggered IRF3 and NF-kappaB activation. Proc Natl Acad Sci U S A **107:**815-820.

11. **Zhong, B., Y. Yang, S. Li, Y. Y. Wang, Y. Li, F. Diao, C. Lei, X. He, L. Zhang, P. Tien, and H. B. Shu.** 2008. The adaptor protein MITA links virus-sensing receptors to IRF3 transcription factor activation. Immunity **29:**538-550.

12. **Ning, Y. J., Q. Mo, K. Feng, Y. Q. Min, M. Y. Li, D. H. Hou, C. Peng, X. Zheng, F. Deng, Z. H. Hu, and H. L. Wang.** 2019. Interferon-gamma-Directed Inhibition of a Novel High-Pathogenic Phlebovirus and Viral Antagonism of the Antiviral Signaling by Targeting STAT1. Front Immunol **10**.

13. **Ning, Y. J., K. Feng, Y. Q. Min, W. C. Cao, M. Wang, F. Deng, Z. Hu, and H. Wang.** 2015. Disruption of type I interferon signaling by the nonstructural protein of severe fever with thrombocytopenia syndrome virus via the hijacking of STAT2 and STAT1 into inclusion bodies. J Virol **89:**4227-4236.

14. **Mo, Q., Z. Xu, F. Deng, H. Wang, and Y.-J. Ning.** 2020. Host restriction of emerging high-pathogenic bunyaviruses via MOV10 by targeting viral nucleoprotein and blocking ribonucleoprotein assembly. PLoS Pathog **16:**e1009129.

15. **Ning, Y. J., K. Feng, Y. Q. Min, F. Deng, Z. H. Hu, and H. L. Wang.** 2017. Heartland virus NSs protein disrupts host defenses by blocking the TBK1 kinase-IRF3 transcription factor interaction and signaling required for interferon induction. J Biol Chem **292:**16722-16733.

16. **Min, Y.-Q., Y.-J. Ning, H. Wang, and F. Deng.** 2020. A RIG-I-like receptor directs antiviral responses to a bunyavirus and is antagonized by virus-induced blockade of TRIM25-mediated ubiquitination. The Journal of biological chemistry **295:**9691-9711.

17. **Hall, E. H., J. L. Balsbaugh, K. L. Rose, J. Shabanowitz, D. F. Hunt, and D. L. Brautigan.** 2010. Comprehensive analysis of phosphorylation sites in Tensin1 reveals regulation by p38MAPK. Mol Cell Proteomics **9:**2853-2863.

18. **Ning, Y.-J., K. Feng, Y.-Q. Min, F. Deng, Z. Hu, and H. Wang.** 2017. Heartland virus NSs protein disrupts host defenses by blocking the TBK1 kinase-IRF3 transcription factor interaction and signaling required for interferon induction. The Journal of biological chemistry **292:**16722-16733.

19. **Belgnaoui, S. M., S. Paz, S. Samuel, M.-L. Goulet, Q. Sun, M. Kikkert, K. Iwai, I. Dikic, J. Hiscott, and R. Lin.** 2012. Linear ubiquitination of NEMO negatively regulates the interferon antiviral response through disruption of the MAVS-TRAF3 complex. Cell Host Microbe **12:**211-222.

20. **Kelstrup, C. D., D. B. Bekker-Jensen, T. N. Arrey, A. Hogrebe, A. Harder, and J. V. Olsen.** 2018. Performance Evaluation of the Q Exactive HF-X for Shotgun Proteomics. J Proteome Res **17:**727-738.

21. **Cox, J., M. Y. Hein, C. A. Luber, I. Paron, N. Nagaraj, and M. Mann.** 2014. Accurate proteome-wide label-free quantification by delayed normalization and maximal peptide ratio extraction, termed MaxLFQ. Mol Cell Proteomics **13:**2513-2526.

22. **TheUniProtConsortium.** 2017. UniProt: the universal protein knowledgebase. Nucleic Acids Res **45:**D158-D169.

23. **Szklarczyk, D., A. L. Gable, D. Lyon, A. Junge, S. Wyder, J. Huerta-Cepas, M. Simonovic, N. T. Doncheva, J. H. Morris, P. Bork, L. J. Jensen, and C. v. Mering.** 2019. STRING v11:

protein-protein association networks with increased coverage, supporting functional discovery in genome-wide experimental datasets. Nucleic Acids Res **47:**D607-D613.

24. **Shannon, P., A. Markiel, O. Ozier, N. S. Baliga, J. T. Wang, D. Ramage, N. Amin, B. Schwikowski, and T. Ideker.** 2003. Cytoscape: a software environment for integrated models of biomolecular interaction networks. Genome Res **13:**2498-2504.
25. **Yu, G., L.-G. Wang, Y. Han, and Q.-Y. He.** 2012. clusterProfiler: an R package for comparing biological themes among gene clusters. Omics **16:**284-287.
26. **Wishart, D. S., Y. D. Feunang, A. C. Guo, E. J. Lo, A. Marcu, J. R. Grant, T. Sajed, D. Johnson, C. Li, Z. Sayeeda, N. Assempour, I. Iynkkaran, Y. Liu, A. Maciejewski, N. Gale, A. Wilson, L. Chin, R. Cummings, D. Le, A. Pon, C. Knox, and M. Wilson.** 2018. DrugBank 5.0: a major update to the DrugBank database for 2018. Nucleic Acids Res **46:**D1074-D1082.

Supplementary figures

Figure S1

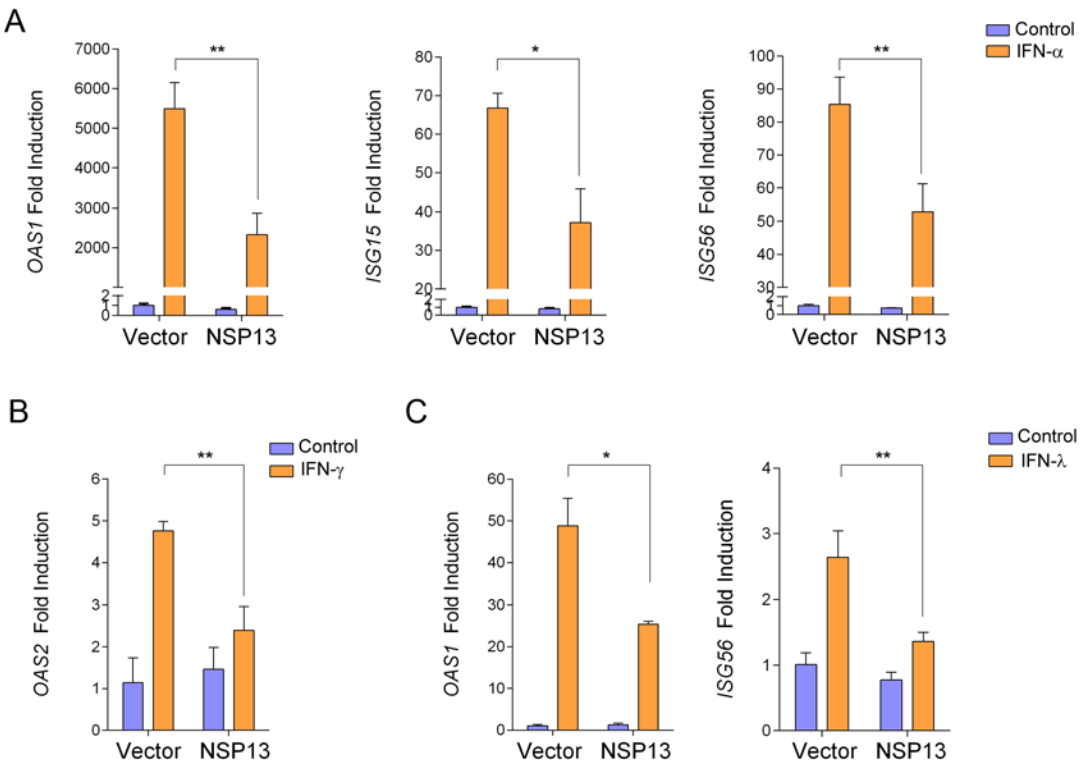

Supplementary Figure S1. SARS-CoV-2 NSP13 inhibits expression of IFN-triggered antiviral genes. HEK293T cells were transfected with control plasmid (vector) or the NSP13 expression plasmid for 24 h and then treated with IFN-α (2000 u/ml) (A), IFN-γ (100 ng/ml) (B), or IFN-λ (200 ng/ml) (C) for 10 h. Expression of representative IFN-stimulated genes (ISGs), including *oligoadenylate synthetase 1* (*OAS1*), *OAS2*, *ISG15*, and *ISG56*, was respectively detected by reverse-transcription quantitative PCR. Data are mean ± SD, $n = 3$. *$p < 0.05$, **$p < 0.01$; unpaired t test or Welch's t test.

Figure S2

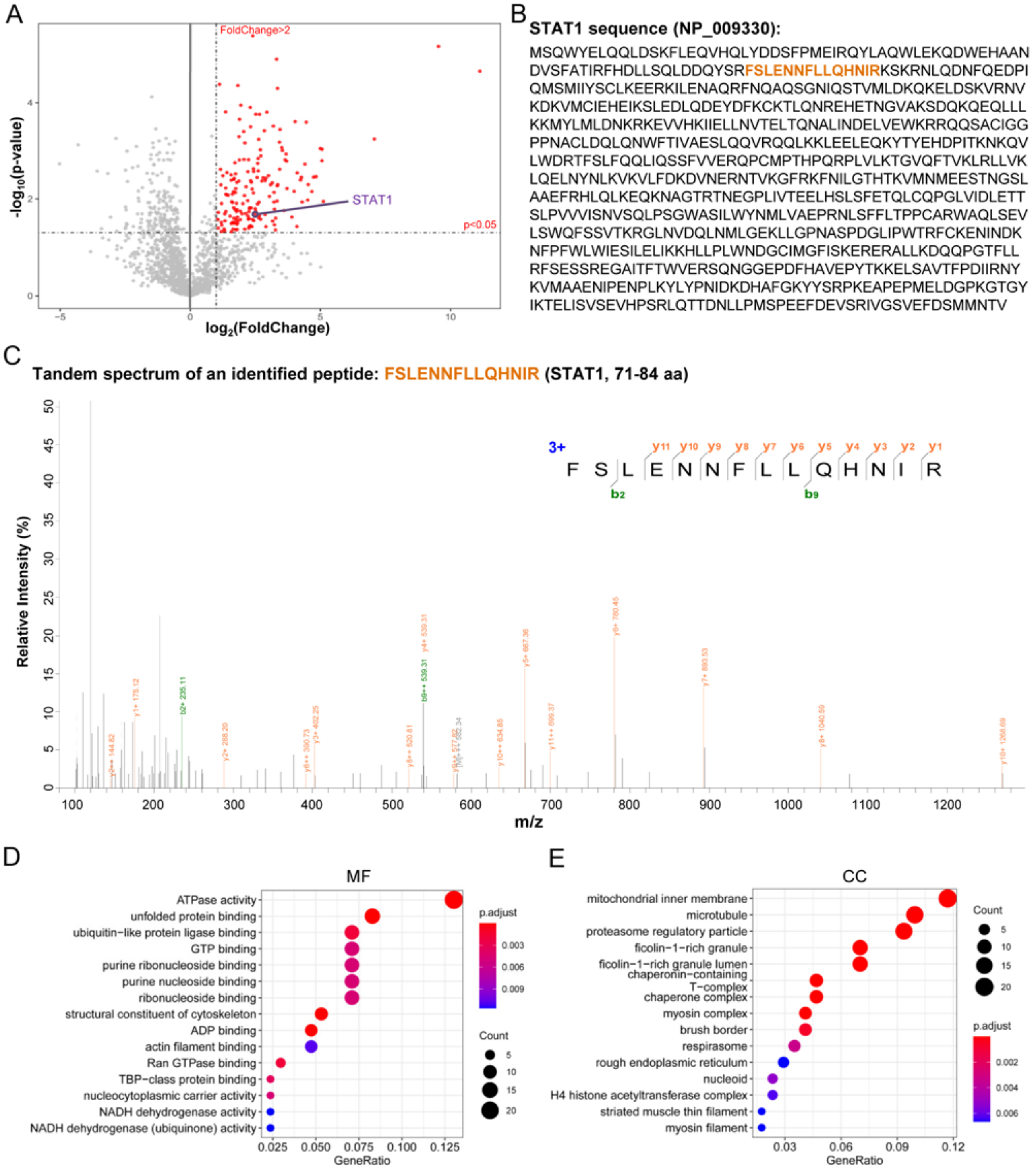

Supplementary Figure S2. Identification of cellular proteins interacting with SARS-CoV-2 NSP13 via MS quantification and bioinformatics analysis. (A) Volcano plot representing MS and statistical analysis results for potential interaction partners of NSP13. Dashed lines indicate the cutoff fold change (2) and p-value (0.05).

Points with a fold change of more than 2 and a p-value of < 0.05 are highlighted in red. STAT1 with a fold change of 5.57 and a p-value of 0.02065 is circled. (B and C) STAT1 peptide identification among NSP13 pulldown products. STAT1 amino acid sequence (B) and representative tandem spectrum of an identified peptide (C) are shown. (D and E) Molecular function (MF) and cellular component (CC) enrichment analyses showing the top 15 enriched gene ontologies for NSP13-interacting proteins. See also Figure 1B-F.

Figure S3

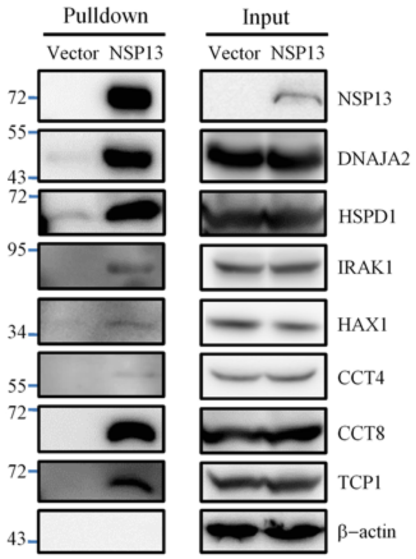

Supplementary Figure S3. Validation of the interactome results using immunoblotting. Interactions of SARS-CoV-2 NSP13 with the indicated endogenous host proteins identified in the interactome analysis were validated using immunoblotting. S-pulldown assays were performed as indicated in Supplementary Materials and Methods. DNAJA2, DnaJ homolog subfamily A member 2; HSPD1, 60 kDa heat shock protein; HAX1, HCLS1-associated protein X-1; IRAK1, interleukin-1 receptor-associated kinase 1; CCT4, T-complex protein 1 subunit delta; CCT8, T-complex protein 1 subunit theta; TCP1, T-complex protein 1 subunit alpha.

Figure S4

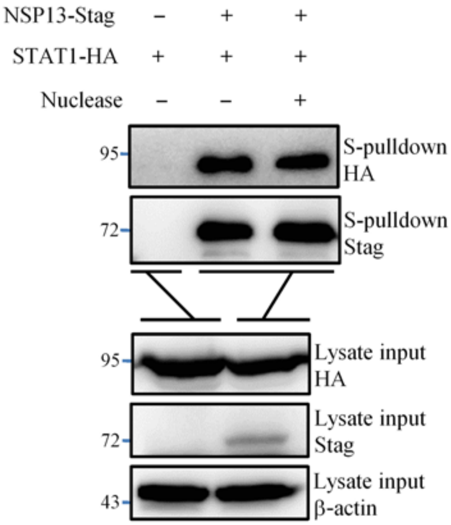

Supplementary Figure S4. NSP13 interacts with STAT1 independently of nucleic acids. HEK293T cells were transfected with the STAT1-HA expression plasmid along with the NSP13-Stag expression plasmid or control. At 36 h post-transfection, cells were lysed for S-pulldown and immunoblot analyses. For the group cotransfected with STAT1 and NSP13 expression plasmids, cell lysate was divided into two and either treated with excessive nuclease (Benzonase, 100 u, Millipore, Cat#70746) or mock treated for 4 h before the pulldown assay.

Figure S5

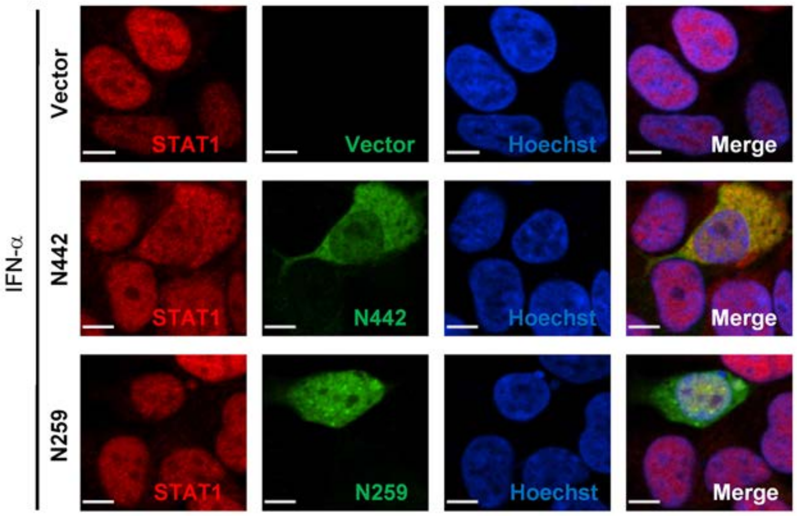

Supplementary Figure S5. Effects of NSP13 truncated mutants on STAT1 nuclear accumulation. HEK293 cells transfected with control plasmid (vector) or expression plasmids encoding HA-tagged NSP13 mutants, N442 or N259, were treated with IFN-α (2000 u/ml) for 30 min, followed by IFA analyses, as in Figure 1M. Bars, 7 μm.

Figure S6

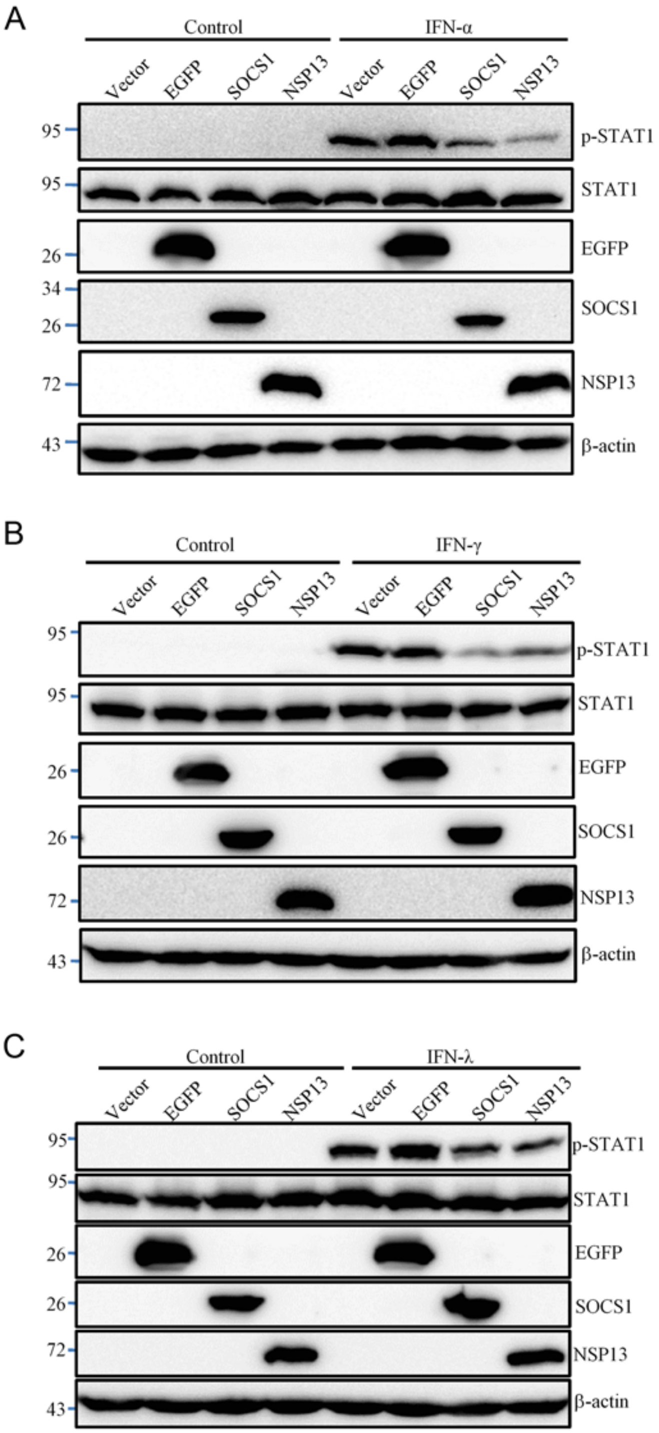

Supplementary Figure S6. Inhibition of STAT1 activation by NSP13 and SOCS1 (but not EGFP). HEK293T cells were transfected with vector plasmid or plasmids encoding EGFP or HA-tagged SOCS1 or NSP13. At 24 h post-transfection, cells were treated with IFN-α (2000 u/ml; A), IFN-γ (100 ng/ml; B), or IFN-λ (200 ng/ml;

C) for 30 min before immunoblotting analyses. SOCS1 and NSP13 expression was detected using an anti-HA antibody; other proteins were detected with specific antibodies, as in Figures 1N, 1Q, and 1R.

Table S1. List of SARS-CoV-2 NSP13-interacting cellular proteins identified in this study.

Accession	Protein	Description	Fold Change	p-Value
Q9BXT6	MOV10L	RNA helicase Mov10l1	2237.39	0.00002
A0A2R8Y7R9	PPFIA1	Liprin-alpha-1 (Fragment)	745.47	0.00000
A0A0S2Z4G6	TPM1	Tropomyosin 1 (Alpha), isoform CRA_o (Fragment)	134.90	0.00057
O60884	DNAJA2	DnaJ homolog subfamily A member 2	34.74	0.01131
Q9BV19	C1orf50	Uncharacterized protein C1orf50	33.51	0.00092
Q96A33	CCDC47	Coiled-coil domain-containing protein 47	33.41	0.00160
Q9Y608	LRRFIP2	Leucine-rich repeat flightless-interacting protein 2	31.94	0.00090
P35579	MYH9	Myosin-9	28.55	0.00344
P10809	HSPD1	60 kDa heat shock protein, mitochondrial	26.70	0.00351
Q53ET0	CRTC2	CREB-regulated transcription coactivator 2	25.46	0.00699
Q7Z406	MYH14	Myosin-14	23.14	0.00501
Q13015	MLLT11	Protein AF1q	22.81	0.01058
P60660	MYL6	Myosin light polypeptide 6	21.92	0.00025
P14649	MYL6B	Myosin light chain 6B	21.18	0.00189
P19105	MYL12A	Myosin regulatory light chain 12A	19.71	0.01163
H0YDN9	ARFGAP	ADP-ribosylation factor GTPase-activating protein 2 (Fragment)	19.30	0.00355
O00148	DDX39A	ATP-dependent RNA helicase DDX39A	18.87	0.00402
P52701	MSH6	DNA mismatch repair protein Msh6	17.32	0.00288
Q9BRT8	CBWD1	COBW domain-containing protein 1	17.18	0.03736
Q9NRF9	POLE3	DNA polymerase epsilon subunit 3	16.36	0.00024
Q7Z6Z7	HUWE1	E3 ubiquitin-protein ligase HUWE1	15.95	0.00694
K7EJE8	LONP1	Lon protease homolog, mitochondrial	14.98	0.02368
P35580	MYH10	Myosin-10	13.15	0.00607
F8VPD4	CAD	Aspartate carbamoyltransferase	13.00	0.00163
Q13885	TUBB2A	Tubulin beta-2A chain	12.84	0.00116
H7BXH2	PPP6R3	Serine/threonine-protein phosphatase 6 regulatory subunit 3	12.52	0.00154
P78527	PRKDC	DNA-dependent protein kinase catalytic subunit	12.14	0.01737
Q96CS3	FAF2	FAS-associated factor 2	11.61	0.00098
A0A5F9UP49	SDF4	45 kDa calcium-binding protein	11.44	0.00085
Q9UJS0	SLC25A	Calcium-binding mitochondrial carrier protein Aralar2	10.90	0.00036
O43164	PJA2	E3 ubiquitin-protein ligase Praja-2	10.80	0.00242
P62195	PSMC5	26S proteasome regulatory subunit 8	10.75	0.00066
Q9BTY7	HGH1	Protein HGH1 homolog	10.26	0.00503
P31689	DNAJA1	DnaJ homolog subfamily A member 1	10.20	0.00005
Q8N1F7	NUP93	Nuclear pore complex protein Nup93	10.13	0.00532
O43242	PSMD3	26S proteasome non-ATPase regulatory subunit 3	10.10	0.01109
Q12965	MYO1E	Unconventional myosin-Ie	9.95	0.00001
P18085	ARF4	ADP-ribosylation factor 4	9.71	0.02493
Q93008	USP9X	Probable ubiquitin carboxyl-terminal hydrolase FAF-X	9.66	0.04425
Q13045	FLII	Protein flightless-1 homolog	9.40	0.01626
O76071	CIAO1	Probable cytosolic iron-sulfur protein assembly protein CIAO1	9.40	0.03452
A0A384DVK	R3HCC1	Coiled-coil domain-containing protein R3HCC1L	9.30	0.00731
O00165	HAX1	HCLS1-associated protein X-1	9.30	0.01163
F8W1Z8	ARL1	ADP-ribosylation factor-like protein 1	9.14	0.00030
A0A0A0MS5	GSN	Actin-depolymerizing factor	8.99	0.02878
Q9UPN7	PPP6R1	Serine/threonine-protein phosphatase 6 regulatory subunit 1	8.88	0.00461
A0A087WXC	NDUFA1	NADH dehydrogenase [ubiquinone] 1 alpha subcomplex subunit 10	8.53	0.00505
Q02978	SLC25A	Mitochondrial 2-oxoglutarate/malate carrier protein	8.26	0.00887
P51570	GALK1	Galactokinase	8.06	0.00328
Q14257	RCN2	Reticulocalbin-2	7.71	0.00015
O14828	SCAMP3	Secretory carrier-associated membrane protein 3	7.52	0.03761

Q9NYL9	TMOD3	Tropomodulin-3	7.51	0.00371
F8WF50	RABL3	Rab-like protein 3	7.36	0.02106
P68371	TUBB4B	Tubulin beta-4B chain	7.35	0.00414
R4GMR5	PSMD8	26S proteasome non-ATPase regulatory subunit 8	7.21	0.00038
Q9BUF5	TUBB6	Tubulin beta-6 chain	6.98	0.00362
Q3ZCQ8	TIMM50	Mitochondrial import inner membrane translocase subunit TIM50	6.75	0.00421
Q5JP53	TUBB	Tubulin beta chain	6.56	0.00127
D3YTB5	IRAK1	Interleukin-1 receptor-associated kinase 1	6.52	0.02645
Q9BVA1	TUBB2B	Tubulin beta-2B chain	6.48	0.00095
Q9UG63	ABCF2	ATP-binding cassette sub-family F member 2	6.33	0.00017
H3BLY2	BORA	Protein aurora borealis (Fragment)	6.32	0.02206
Q15293	RCN1	Reticulocalbin-1	6.25	0.01170
P78318	IGBP1	Immunoglobulin-binding protein 1	6.18	0.02454
C9JQ00	TUBA4A	Tubulin alpha chain (Fragment)	6.18	0.00057
O00743	PPP6C	Serine/threonine-protein phosphatase 6 catalytic subunit	6.11	0.00262
Q96P70	IPO9	Importin-9	6.09	0.00144
P43686	PSMC4	26S proteasome regulatory subunit 6B	6.03	0.00182
E9PLD2	MTFR1L	Mitochondrial fission regulator 1-like	6.00	0.01272
O95573	ACSL3	Long-chain-fatty-acid--CoA ligase 3	5.94	0.00357
O00232	PSMD12	26S proteasome non-ATPase regulatory subunit 12	5.73	0.00017
Q9NR33	POLE4	DNA polymerase epsilon subunit 4	5.73	0.01815
Q9Y5L4	TIMM13	Mitochondrial import inner membrane translocase subunit Tim13	5.64	0.01589
**E7ENM1**	**STAT1**	**Signal transducer and activator of transcription 1**	**5.57**	**0.02065**
Q8N0X7	SPART	Spartin	5.42	0.02812
O43852	CALU	Calumenin	5.40	0.00270
P43246	MSH2	DNA mismatch repair protein Msh2	5.37	0.00236
Q9Y3D0	CIAO2B	Cytosolic iron-sulfur assembly component 2B	5.37	0.00750
B0YIW6	ARCN1	Coatomer subunit delta	5.28	0.00000
O00487	PSMD14	26S proteasome non-ATPase regulatory subunit 14	5.25	0.02675
Q7Z5K2	WAPL	Wings apart-like protein homolog	5.19	0.00621
Q9HCN8	SDF2L1	Stromal cell-derived factor 2-like protein 1	5.16	0.03766
Q9UQE7	SMC3	Structural maintenance of chromosomes protein 3	5.08	0.00447
Q9ULX3	NOB1	RNA-binding protein NOB1	5.05	0.01530
Q9UNM6	PSMD13	26S proteasome non-ATPase regulatory subunit 13	4.99	0.00415
P12236	SLC25A	ADP/ATP translocase 3	4.98	0.00031
Q5SRD1	TIMM23	Mitochondrial import inner membrane translocase subunit Tim23B	4.97	0.03613
P62191	PSMC1	26S proteasome regulatory subunit 4	4.93	0.00012
P04350	TUBB4A	Tubulin beta-4A chain	4.88	0.01512
C9JA28	SSR3	Signal sequence receptor subunit gamma	4.83	0.01567
P43307	SSR1	Translocon-associated protein subunit alpha	4.83	0.03696
Q969F1	GTF3C6	General transcription factor 3C polypeptide 6	4.79	0.02558
Q9NVI7	ATAD3A	ATPase family AAA domain-containing protein 3A	4.77	0.01824
A0A2R8Y7U1	TPP1	Tripeptidyl aminopeptidase (Fragment)	4.76	0.04038
P05141	SLC25A	ADP/ATP translocase 2	4.71	0.00153
Q6P1M0	SLC27A	Long-chain fatty acid transport protein 4	4.69	0.02635
Q96IX5	ATP5MD	ATP synthase membrane subunit DAPIT, mitochondrial	4.64	0.02260
O14980	XPO1	Exportin-1	4.63	0.00272
P55036	PSMD4	26S proteasome non-ATPase regulatory subunit 4	4.60	0.01086
Q9Y265	RUVBL1	RuvB-like 1	4.43	0.00556
Q9P2I0	CPSF2	Cleavage and polyadenylation specificity factor subunit 2	4.39	0.02001
Q99460	PSMD1	26S proteasome non-ATPase regulatory subunit 1	4.28	0.00068
Q8NFH3	NUP43	Nucleoporin Nup43	4.27	0.04252
P07919	UQCRH	Cytochrome b-c1 complex subunit 6, mitochondrial	4.24	0.00852
O00231	PSMD11	26S proteasome non-ATPase regulatory subunit 11	4.19	0.00151

Q9H9B4	SFXN1	Sideroflexin-1	4.16	0.00039
E9PID8	CSTF2	Cleavage stimulation factor subunit 2	4.07	0.01250
Q99470	SDF2	Stromal cell-derived factor 2	3.98	0.02026
Q9NS69	TOMM2	Mitochondrial import receptor subunit TOM22 homolog	3.85	0.00011
F8VWL0	TFCP2	Alpha-globin transcription factor CP2 (Fragment)	3.81	0.01800
Q14671	PUM1	Pumilio homolog 1	3.80	0.02691
Q9P0J0	NDUFA1	NADH dehydrogenase [ubiquinone] 1 alpha subcomplex subunit 13	3.72	0.00022
P30153	PPP2R1	Serine/threonine-protein phosphatase 2A 65 kDa regulatory subunit	3.71	0.00155
P68363	TUBA1B	Tubulin alpha-1B chain	3.67	0.00050
O43592	XPOT	Exportin-T	3.65	0.00422
Q2TAL8	QRICH1	Glutamine-rich protein 1	3.56	0.00004
Q9Y230	RUVBL2	RuvB-like 2	3.53	0.02837
P51665	PSMD7	26S proteasome non-ATPase regulatory subunit 7	3.52	0.02380
U3KQG0	TARS2	Threonyl-tRNA synthetase	3.52	0.03154
P16615	ATP2A2	Sarcoplasmic/endoplasmic reticulum calcium ATPase 2	3.50	0.01669
A0A0C4DGW	C5orf51	UPF0600 protein C5orf51	3.49	0.04328
P50402	EMD	Emerin	3.48	0.03348
P50991	CCT4	T-complex protein 1 subunit delta	3.47	0.00452
P14406	COX7A2	Cytochrome c oxidase subunit 7A2, mitochondrial	3.43	0.01234
P11172	UMPS	Uridine 5-monophosphate synthase	3.43	0.01813
Q96EY1	DNAJA3	DnaJ homolog subfamily A member 3, mitochondrial	3.37	0.02688
A0A087WWU	TPM3	Tropomyosin alpha-3 chain	3.36	0.00610
C9JYE9	E2F5	Transcription factor E2F5 (Fragment)	3.35	0.04360
O14654	IRS4	Insulin receptor substrate 4	3.34	0.00302
E7ENT5	SLC39A	Zinc transporter ZIP10 (Fragment)	3.33	0.00737
Q9H4I9	SMDT1	Essential MCU regulator, mitochondrial	3.30	0.01654
Q99832	CCT7	T-complex protein 1 subunit eta	3.29	0.00177
A0A087X2B5	BSG	Basigin (Fragment)	3.23	0.00374
H3BRE8	RPAP1	RNA polymerase II-associated protein 1	3.22	0.03077
P49411	TUFM	Elongation factor Tu, mitochondrial	3.21	0.02492
H7BZ50	MZT2B	Mitotic-spindle organizing protein 2B (Fragment)	3.20	0.03205
Q9UNF1	MAGED	Melanoma-associated antigen D2	3.19	0.01513
S4R3N9	SFXN3	Sideroflexin-3	3.18	0.00392
P55060	CSE1L	Exportin-2	3.17	0.00706
O43347	MSI1	RNA-binding protein Musashi homolog 1	3.13	0.04244
P56385	ATP5ME	ATP synthase subunit e, mitochondrial	3.09	0.03057
A0A087X1G1	NDUFA5	Complex I subunit B13	3.09	0.01189
Q969M3	YIPF5	Protein YIPF5	3.08	0.00796
P50990	CCT8	T-complex protein 1 subunit theta	3.03	0.01147
Q15008	PSMD6	26S proteasome non-ATPase regulatory subunit 6	3.00	0.04804
Q96PU8	QKI	Protein quaking	2.99	0.03248
Q53EZ4	CEP55	Centrosomal protein of 55 kDa	2.98	0.00405
B4DUR8	CCT3	T-complex protein 1 subunit gamma	2.97	0.00316
O00273	DFFA	DNA fragmentation factor subunit alpha	2.89	0.02268
Q92973	TNPO1	Transportin-1	2.74	0.00835
P40227	CCT6A	T-complex protein 1 subunit zeta	2.65	0.00283
C9JXK0	LBR	Delta(14)-sterol reductase LBR (Fragment)	2.63	0.00819
P78371	CCT2	T-complex protein 1 subunit beta	2.59	0.00799
O75794	CDC123	Cell division cycle protein 123 homolog	2.58	0.01816
Q9UBI1	COMMD	COMM domain-containing protein 3	2.57	0.00592
P17987	TCP1	T-complex protein 1 subunit alpha	2.56	0.00015
Q92616	GCN1	eIF-2-alpha kinase activator GCN1	2.52	0.02845
P48643	CCT5	T-complex protein 1 subunit epsilon	2.50	0.02035
E9PFD4	BZW2	BZW2 protein	2.49	0.04604

S4R3B5	SEC61B	Protein transport protein Sec61 subunit beta	2.46	0.04226
A0A2R8Y653	UCK2	Uridine kinase	2.38	0.00330
P62333	PSMC6	26S proteasome regulatory subunit 10B	2.31	0.03510
O75489	NDUFS3	NADH dehydrogenase [ubiquinone] iron-sulfur protein 3,	2.30	0.01244
J3KN29	PSMD9	26S proteasome non-ATPase regulatory subunit 9	2.29	0.04738
P04844	RPN2	Dolichyl-diphosphooligosaccharide-protein glycosyltransferase	2.28	0.03061
Q53GQ0	HSD17B	Very-long-chain 3-oxoacyl-CoA reductase	2.25	0.00507
P05023	ATP1A1	Sodium/potassium-transporting ATPase subunit alpha-1	2.23	0.00243
P25398	RPS12	40S ribosomal protein S12	2.18	0.00004
P04843	RPN1	Dolichyl-diphosphooligosaccharide-protein glycosyltransferase	2.10	0.01899
O95400	CD2BP2	CD2 antigen cytoplasmic tail-binding protein 2	2.10	0.04033
R4GNH3	PSMC3	26S proteasome regulatory subunit 6A	2.08	0.02685
B1ALA9	PRPS1	Ribose-phosphate pyrophosphokinase 1	2.06	0.02682

Table S2. List of druggable cellular targets interacting with NSP13 and the targeting drugs.

Target	DrugBank ID	Drug Name	Group
TPM1	DB12695	Phenethyl Isothiocyanate	Investigational
TPM1	DB11638	Artenimol	Experimental
MYH9	DB11638	Artenimol	Experimental
HSPD1	DB09130	Copper	Approved
MYH14	DB01944	(S)-blebbistatin	Experimental
MYH14	DB03126	Mant-Adp	Experimental
MYH14	DB07470	(3aS)-3a-hydroxy-1-phenyl-1,2,3,3a-tetrahydro-4H-pyrrolo[2,3-b] quinolin-4-one	Experimental
MYH14	DB08276	trifluoro-[hydroxy-[hydroxy-[2-(N-methyl-2-nitro-anilino) ethoxy]phosphoryl]oxy-phosphoryl] oxy-beryllium(1-)	Experimental
MYH14	DB04444	Tetrafluoroaluminate Ion	Experimental
MYH14	DB07468	(3aS)-3a-hydroxy-5-methyl-1-phenyl-1,2,3,3a-tetrahydro-4H -pyrrolo[2,3-b]quinolin-4-one	Experimental
MYH14	DB07469	(3aS)-3a-hydroxy-7-methyl-1-phenyl-1,2,3,3a-tetrahydro-4H-pyrrolo[2 ,3-b]quinolin-4-one	Experimental
MYL6B	DB08378	4-[4-(2,5-DIOXO-PYRROLIDIN-1-YL)-PHENYLAMINO]-4-HYDR OXY-BUTYRIC ACID	Experimental
MYL12A	DB08378	4-[4-(2,5-DIOXO-PYRROLIDIN-1-YL)-PHENYLAMINO]-4-HYDR OXY-BUTYRIC ACID	Experimental
POLE3	DB00242	Cladribine	Approved
CAD	DB00128	Aspartic acid	Approved
CAD	DB03459	Sparfosic acid	Experimental
TUBB2A	DB12695	Phenethyl Isothiocyanate	Investigational
TUBB2A	DB05147	CYT997	Investigational
PRKDC	DB00201	Caffeine	Approved
PRKDC	DB05210	SF1126	Investigational
SLC25A13	DB00128	Aspartic acid	Approved
MYO1E	DB03366	Imidazole	Experimental
ARF4	DB04315	Guanosine-5'-Diphosphate	Experimental
ARL1	DB04315	Guanosine-5'-Diphosphate	Experimental
GSN	DB09130	Copper	Approved
GSN	DB02621	Latrunculin A	Experimental
GSN	DB14487	Zinc acetate	Approved
GSN	DB01593	Zinc	Approved
NDUFA10	DB00157	NADH	Approved
GALK1	DB04395	Phosphoaminophosphonic Acid-Adenylate Ester	Experimental
TUBB4B	DB00518	Albendazole	Approved
TUBB4B	DB04910	Oxibendazole	Investigational
TUBB4B	DB12695	Phenethyl Isothiocyanate	Investigational
TUBB4B	DB03010	Patupilone	Experimental
TUBB4B	DB05147	CYT997	Investigational
TUBB4B	DB01873	Epothilone D	Investigational

TUBB4B	DB00643	Mebendazole	Approved
TUBB6	DB11638	Artenimol	Experimental
TUBB6	DB05147	CYT997	Investigational
TUBB	DB01179	Podofilox	Approved
TUBB	DB00570	Vinblastine	Approved
TUBB	DB01394	Colchicine	Approved
TUBB	DB00541	Vincristine	Approved
TUBB	DB12334	Milataxel	Investigational
TUBB	DB05284	CA4P	Investigational
TUBB	DB11731	Depatuxizumab mafodotin	Investigational
TUBB	DB09130	Copper	Approved
TUBB	DB15534	Colchiceine	Approved
TUBB	DB11638	Artenimol	Experimental
TUBB	DB03010	Patupilone	Experimental
TUBB	DB05147	CYT997	Investigational
TUBB	DB06042	ZEN-012	Investigational
TUBB	DB11641	Vinflunine	Approved
TUBB	DB01873	Epothilone D	Investigational
TUBB	DB00361	Vinorelbine	Approved
IRAK1	DB12010	Fostamatinib	Approved
TUBB2B	DB05147	CYT997	Investigational
TUBB2B	DB07574	2-MERCAPTO-N-[1,2,3,10-TETRAMETHOXY-9-OXO-5,6,7,9-TETRAHYDRO-BENZO[A]HEPTALEN-7-YL]ACETAMIDE	Experimental
TUBA4A	DB01179	Podofilox	Approved
TUBA4A	DB00541	Vincristine	Approved
TUBA4A	DB12695	Phenethyl Isothiocyanate	Investigational
TUBA4A	DB03010	Patupilone	Experimental
TUBA4A	DB05147	CYT997	Investigational
TUBA4A	DB06772	Cabazitaxel	Approved
TUBA4A	DB01873	Epothilone D	Investigational
POLE4	DB00242	Cladribine	Approved
SLC25A6	DB00720	Clodronic acid	Approved
SLC25A6	DB01077	Etidronic acid	Approved
TUBB4A	DB11638	Artenimol	Experimental
TUBB4A	DB03010	Patupilone	Experimental
TUBB4A	DB05147	CYT997	Investigational
TUBB4A	DB06042	ZEN-012	Investigational
TUBB4A	DB01873	Epothilone D	Investigational
SLC25A5	DB00720	Clodronic acid	Approved
SLC25A5	DB01077	Etidronic acid	Approved
XPO1	DB11942	Selinexor	Approved
UQCRH	DB08690	Ubiquinone Q2	Experimental
UQCRH	DB07778	(S)-famoxadone	Experimental
UQCRH	DB04141	2-Hexyloxy-6-Hydroxymethyl- Tetrahydro-Pyran-3,4,5-Triol	Experimental

UQCRH	DB07763	(5S)-3-ANILINO-5-(2,4-DIFLUOROPHENYL)-5-METHYL-1,3-OXAZOLIDINE-2,4-DIONE	Experimental
UQCRH	DB08453	2-Nonyl-4-quinolinol 1-oxide	Experimental
UQCRH	DB08330	METHYL(2Z)-3-METHOXY-2-{2-[(E)-2-PHENYLVINYL] PHENYL}ACRYLATE	Experimental
UQCRH	DB04799	6-Hydroxy-5-undecyl-4,7-benzothiazoledione	Experimental
UQCRH	DB07401	Azoxystrobin	Experimental
SDF2	DB02201	Malonate Ion	Experimental
NDUFA13	DB00157	NADH	Approved
PPP2R1A	DB02506	2,6,8-Trimethyl-3-Amino-9-Benzyl-9-Methoxynonanoic Acid	Experimental
PPP2R1A	DB06905	(2S,3S,4E,6E,8S,9S)-3-amino-9-methoxy-2,6,8-trimethyl-10-phenyldeca-4,6-dienoic acid	Experimental
TUBA1B	DB03010	Patupilone	Experimental
TUBA1B	DB05147	CYT997	Investigational
TUBA1B	DB07574	2-MERCAPTO-N-[1,2,3,10-TETRAMETHOXY-9-OXO-5,6,7,9-TETRAHYDRO-BENZO[A]HEPTALEN -7-YL]ACETAMIDE	Experimental
TUBA1B	DB01873	Epothilone D	Investigational
RUVBL2	DB04216	Quercetin	Experimental
TARS2	DB00156	Threonine	Approved
ATP2A2	DB06157	Istaroxime	Investigational
UMPS	DB02890	6-hydroxyuridine-5'-phosphate	Experimental
TPM3	DB12695	Phenethyl Isothiocyanate	Investigational
TUFM	DB04315	Guanosine-5'-Diphosphate	Experimental
TUFM	DB14533	Zinc chloride	Approved
TUFM	DB14548	Zinc sulfate, unspecified form	Approved
TUFM	DB14487	Zinc acetate	Approved
TUFM	DB01593	Zinc	Approved
NDUFA5	DB00157	NADH	Approved
CCT3	DB04395	Phosphoaminophosphonic Acid-Adenylate Ester	Experimental
CCT3	DB11638	Artenimol	Experimental
UCK2	DB02097	Cytidine	Experimental
UCK2	DB04272	Citric acid	Approved
UCK2	DB02431	Cytidine-5'-Triphosphate	Experimental
UCK2	DB03403	Cytidine-5'-Monophosphate	Experimental
UCK2	DB04005	Uridine 5'-triphosphate	Experimental
NDUFS3	DB00157	NADH	Approved
ATP1A1	DB01021	Trichlormethiazide	Approved
ATP1A1	DB01244	Bepridil	Approved
ATP1A1	DB00511	Acetyldigitoxin	Approved
ATP1A1	DB01430	Almitrine	Approved
ATP1A1	DB01078	Deslanoside	Approved
ATP1A1	DB01119	Diazoxide	Approved
ATP1A1	DB00774	Hydroflumethiazide	Approved
ATP1A1	DB01396	Digitoxin	Approved

ATP1A1	DB14515	Magnesium lactate	Nutraceutical
ATP1A1	DB14514	Magnesium levulinate	Nutraceutical
ATP1A1	DB13996	Magnesium acetate	Approved
ATP1A1	DB14517	Aluminium phosphate	Approved
ATP1A1	DB14498	Potassium acetate	Approved
ATP1A1	DB06157	Istaroxime	Investigational
ATP1A1	DB14499	Potassium sulfate	Approved
ATP1A1	DB14518	Aluminum acetate	Approved
ATP1A1	DB14500	Potassium	Approved
ATP1A1	DB00390	Digoxin	Approved
ATP1A1	DB00903	Etacrynic acid	Approved
ATP1A1	DB01188	Ciclopirox	Approved
ATP1A1	DB01345	Potassium cation	Approved
ATP1A1	DB01370	Aluminium	Approved
ATP1A1	DB01378	Magnesium cation	Approved
ATP1A1	DB01158	Bretylium	Approved
ATP1A1	DB01092	Ouabain	Approved
ATP1A1	DB09479	Rubidium Rb-82	Approved
ATP1A1	DB13620	Potassium gluconate	Approved
ATP1A1	DB13749	Magnesium gluconate	Approved
PSMC3	DB12695	Phenethyl Isothiocyanate	Investigational
